# Insights into the Identification of iPSC- and Monocyte-Derived Macrophage-Polarizing Compounds by AI-Fueled Cell Painting Analysis Tools

**DOI:** 10.3390/ijms252212330

**Published:** 2024-11-17

**Authors:** Johanna B. Brüggenthies, Jakob Dittmer, Eva Martin, Igor Zingman, Ibrahim Tabet, Helga Bronner, Sarah Groetzner, Julia Sauer, Mozhgan Dehghan Harati, Rebekka Scharnowski, Julia Bakker, Katharina Riegger, Caroline Heinzelmann, Birgit Ast, Robert Ries, Sophie A. Fillon, Anna Bachmayr-Heyda, Kerstin Kitt, Marc A. Grundl, Ralf Heilker, Lina Humbeck, Michael Schuler, Bernd Weigle

**Affiliations:** 1 Department Cancer Immunology and Immune Modulation, Boehringer Ingelheim Pharma GmbH & Co. KG, 88397 Biberach a.d. Riss, Germany; johanna_barbara.brueggenthies@boehringer-ingelheim.com (J.B.B.); rebekka.scharnowski@boehringer-ingelheim.com (R.S.); julia.bakker@boehringer-ingelheim.com (J.B.); katharina.riegger@boehringer-ingelheim.com (K.R.); kerstin.kitt@boehringer-ingelheim.com (K.K.); 2 Department Cancer Immunology and Immune Modulation, Boehringer Ingelheim RCV GmbH & Co. KG, 1121 Vienna, Austria; jakob.dittmer@boehringer-ingelheim.com (J.D.); anna.bachmayr-heyda@boehringer-ingelheim.com (A.B.-H.); 3 Global Drug Discovery Sciences, Boehringer Ingelheim Pharma GmbH & Co. KG, 88397 Biberach a.d. Riss, Germany; eva.martin@boehringer-ingelheim.com (E.M.); helga.bronner@boehringer-ingelheim.com (H.B.); mozhgan.dehghan_harati@boehringer-ingelheim.com (M.D.H.); robert.ries@boehringer-ingelheim.com (R.R.); ralf.heilker@boehringer-ingelheim.com (R.H.); michael.schuler@boehringer-ingelheim.com (M.S.); 4 Global Medicinal Chemistry, Boehringer Ingelheim Pharma GmbH & Co. KG, 88397 Biberach a.d. Riss, Germany; igor.zingman@mpl.mpg.de (I.Z.); marc.grundl@boehringer-ingelheim.com (M.A.G.); lina.humbeck@boehringer-ingelheim.com (L.H.); 5 ScreeningHub und ValueData GmbH, 70563 Stuttgart, Germany; i.tabet@valuedata.io (I.T.); c.heinzelmann@valuedata.io (C.H.); b.ast@valuedata.io (B.A.); 6 Department Immunology and Respiratory, Boehringer Ingelheim Pharma GmbH & Co. KG, 88397 Biberach a.d. Riss, Germany; sarah.groetzner@boehringer-ingelheim.com (S.G.); julia.sauer@boehringer-ingelheim.com (J.S.); 7 Department Cancer Immunology and Immune Modulation, Boehringer Ingelheim Pharmaceuticals, Inc., Ridgefield, CT 06877, USA; sophie.fillon@boehringer-ingelheim.com

**Keywords:** macrophage (re-)polarization, feature extraction, macrophage plasticity, human iPSC, high-content imaging, cell painting, deep learning, macrophage-polarizing compounds, phenotypes, artificial intelligence-fueled image analysis

## Abstract

Macrophage polarization critically contributes to a multitude of human pathologies. Hence, modulating macrophage polarization is a promising approach with enormous therapeutic potential. Macrophages are characterized by a remarkable functional and phenotypic plasticity, with pro-inflammatory (M1) and anti-inflammatory (M2) states at the extremes of a multidimensional polarization spectrum. Cell morphology is a major indicator for macrophage activation, describing M1(-like) (rounded) and M2(-like) (elongated) states by different cell shapes. Here, we introduced cell painting of macrophages to better reflect their multifaceted plasticity and associated phenotypes beyond the rigid dichotomous M1/M2 classification. Using high-content imaging, we established deep learning- and feature-based cell painting image analysis tools to elucidate cellular fingerprints that inform about subtle phenotypes of human blood monocyte-derived and iPSC-derived macrophages that are characterized as screening surrogate. Moreover, we show that cell painting feature profiling is suitable for identifying inter-donor variance to describe the relevance of the morphology feature ‘cell roundness’ and dissect distinct macrophage polarization signatures after stimulation with known biological or small-molecule modulators of macrophage (re-)polarization. Our novel established AI-fueled cell painting analysis tools provide a resource for high-content-based drug screening and candidate profiling, which set the stage for identifying novel modulators for macrophage (re-)polarization in health and disease.

## 1. Introduction

Macrophages (MΦs) are innate immune cells characterized by high functional and phenotypic plasticity with the classically activated pro-inflammatory (M1) and the alternatively activated anti-inflammatory (M2) states at the extreme ends of a multidimensional polarization spectrum [1,2,3,4,5]. Owing to their critical function in pathogen defense, immune regulation, tissue homeostasis, and repair [6], MΦ polarization has been implicated in critically contributing to many human diseases including cancer, fibrosis, and obesity, as well as cardiovascular, inflammatory, and neurodegenerative diseases [7,8,9,10]. Hence, modulating MΦ polarization is a promising therapeutic approach to address the development or progression of many diseases [9]. Accordingly, much effort in drug discovery is currently devoted to the identification of small-molecule compounds (cpds) with MΦ-modulating, -polarizing, and -reprogramming capacity.

Monocyte-derived MΦs (MDMs) generated from human blood monocytes have been used as a versatile in vitro surrogate of tissue-resident MΦs for decades [11,12,13,14,15]. However, the limited availability of monocytes obtained from blood donations as well as their functional and phenotypic heterogeneity due to inter-donor variance [16] severely hampers their utilization for large-scale high-throughput cpd screening and drug candidate profiling [17]. We and others have recently shown that human-induced pluripotent stem cells (iPSC) can be differentiated towards iPSC-derived MΦs (IDMs) that functionally resemble MDMs [18,19,20,21,22,23,24,25,26].

With the aim to overcome the limitations associated with MDMs in drug discovery, we refined two currently published 201B7 line-based IDM protocols that lead to the generation of tissue-resident-like IDMs via the monocyte- (CD14+ progenitors) or the hematopoietic stem cell-like (CD34+ progenitors) route [18,19]. Here, we characterize the IDMs compared with MDMs isolated and purified from frozen PBMC stocks from several donors in terms of their surface marker expression, their freezing–thawing recovery rate, as well as their large-scale production suitable for screening and profiling purposes.

Due to the non-dichotomous plasticity of MΦs, efforts to characterize their polarization by cell surface receptor markers (e.g., CD80, CD86, CD163, CD206), cytokines (e.g., IL-10), chemokines (e.g., CCL17), and enzymes (e.g., arginase) have been challenged [3,27,28,29], especially in human systems [17]. Based on the correlation between cell morphology and MΦ activation, MΦ shape has been suggested as a promising indicator to describe the polarization of human MDMs and murine bone marrow-derived MΦs (BMDMs) [30]. Specifically, M1(-like) MDMs and BMDMs were reported to appear round and flattened, whereas M2(-like) cells are present with an elongated morphology [30,31,32]. In this context, cell shape change has been implemented as a promising read-out in high-content imaging combined with high-throughput screening for the identification of MDM-polarizing cpds [30]. Here, we develop a semi-automated high-content imaged-based analysis tool to evaluate the shape changes of IDMs in comparison with MDMs as a valid measurement of MΦ polarization and reprogramming. We address the phenotypic spectrum of biological- and cpd-stimulated IDMs and MDMs and underline the impact and relevance of the cell roundness as screening read-out. To clearly state the MΦ polarization terminology, we referred to M1- or M2-like for compound induction and M1 or M2 for biological stimulation.

Recently, cell painting [33,34,35,36,37] has gained increasing attention in drug discovery as an image-based approach to perform phenotypic profiling of human cell lines for describing disease-associated phenotypes [38,39], understanding disease mechanisms [40], and predicting drug properties such as mode of action (MoA), cytotoxicity, off-target effects, and bioactivity [38,41,42,43,44,45]. Cell painting is a cost-efficient morphological profiling assay that multiplexes six fluorescent dyes imaged in five channels to reveal eight organelles or cell compartments (DNA, cytoplasmic RNA, nuclei, actin filaments, Golgi apparatus, plasma membrane, ER, and mitochondria). Thereby, thousands of phenotypic features are obtained that inform us about the cellular state [35]. Combining cell painting with computational biology [46,47,48,49,50], the automated image data analysis pipeline can comprise parallel multi-parameter clustering via the identification of single cells and the processing of thousands of their individual features [33]. Consequently, cell painting is broadly applicable across disease-relevant disorders and drug target modalities [51]: it opens a new avenue in phenotypic drug discovery for the early safety and off-target assessment of cpds [52,53], for boosting the lead identification rate and diversity of screening libraries [43], as well as for the evaluation of cellular fingerprints across different cellular screening model systems [54,55,56,57]. Therefore, we reasoned that cell painting can be adapted to generate feature signatures reflecting the multidimensional plasticity of MΦs beyond the traditional M1/M2 paradigm. Here, we establish cell painting of human IDMs (201B7 and ChiPSC12 line) and MDMs (several donors) and describe the high-content cell painting assay in combination with our refined IDM protocol as a scalable and versatile approach to describe MΦ-polarizing cpds and phenotypes. Moreover, we establish an artificial intelligence (AI)-fueled image analysis platform, consisting of a deep learning (DL)- and a feature-based analysis tool for drug screening purposes that enables the identification and quantification of MΦ polarization and reprogramming effects modulated by known biological and cpd stimuli, which can be used across both MΦ systems. In this context, we highlight the relevance of morphological properties such as the ‘cell roundness feature’ to describe MΦ (re-)polarization conditions. In summary, we present novel analysis tools for the description of MΦ (re-)polarization effects by phenotypic cell painting feature fingerprints using high-content imaging combined with deep learning and feature extraction.

## 2. Results

### 2.1. Frozen iPSC-Derived MΦs Are Suitable as a Screening Surrogate of Monocyte-Derived MΦs Generated from Frozen PBMCs

**Figure 1:**
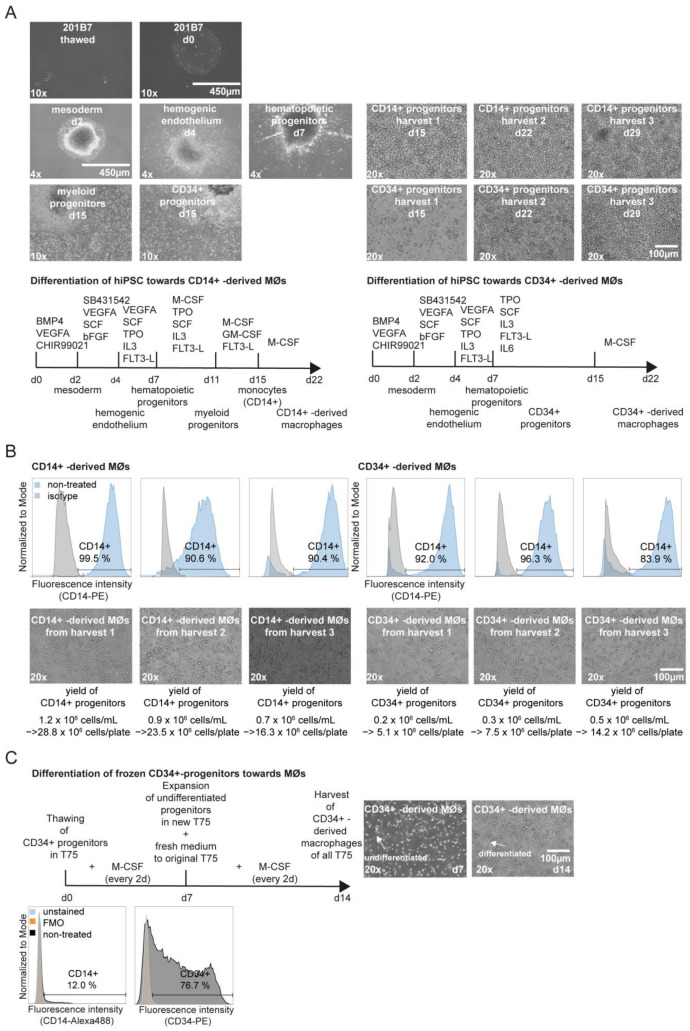
IDM generation from 201B7 via CD14+ or CD34+ progenitors. (**A**) Schematic of the used differentiation protocols to generate CD34+- or CD14+-derived MΦs from the hiPSC line 201B7. Representative brightfield images illustrate the differentiation stages over 22 days. (**B**) Flow cytometry analysis of CD14 expression of CD34+- versus CD14+-derived MΦs across harvest rounds 1–3 (in %; *n* = 3; gating is based on a 1% isotype control). The surface marker expression of CD14 was tracked to verify the differentiation of CD34+ progenitors and CD14+ progenitors towards MΦs. Yield comparison of produced CD34+ and CD14+ progenitors from each harvest (*n* = 3). (**C**) Representative brightfield images illustrate frozen CD34+-derived MΦ differentiation from day 7 (d7) to day 14 (d14). Schematic of the used differentiation protocol of frozen CD34+ progenitors towards IDMs. Flow cytometry analysis of CD14 and CD34 expression of frozen CD34+-derived progenitors (in %; *n* = 3; gating is based on a 1% isotype control).

The iPSC line 201B7 is generated by the retroviral transduction of human fibroblasts and grows in a colony format [20]. Recent multi-omics and functional studies on efferocytosis, phagocytosis, and cytokine release [18,19,26] highlighted the potential of cell line 201B7 to investigate iPSC-derived MΦs. After confirming the pluripotency of the 201B7 cells (Figure S1A), we differentiated these iPSC colonies (size range of 400 to 750 μm) from mesoderm towards hemogenic endothelium into hematopoietic progenitor cells that are floating around the organoid (Figure 1A). At this stage, we decided to further elaborate on two published 201B7-based differentiation protocols [18,19] that vary in the induction step where CD14+ monocytes [18] or CD34+ progenitors [19] are derived from the hematopoietic floaters at day 7 (Figure 1A). Specific surface marker expression (lymphocyte marker CD45, monocytic lineage marker CD14, hematopoietic stem cell marker CD34, MΦ markers CD206, CD163, and CD80) was tracked at the final stage of both progenitor types with 82.0% to 98.9% purity of CD34 or CD14 expression, respectively (Figure S1B).

During early ontogeny and throughout adult life, tissue-resident MΦs are generated from different origins, starting from the yolk sac to the fetal liver, and after birth, from the bone marrow [1,12,13,58]. Thereby, tissue-resident MΦs are not only continuously replaced by monocytes (CD14+ progenitors) recruited from the peripheral blood but established by hematopoietic precursor cells that seed the tissues during embryonic hematopoiesis and complete their differentiation self-maintained across adulthood in the tissue-site [13,59,60]. To compare adult and tissue-resident-like MΦs derived from blood circulating monocytes (CD14+ progenitors) with hematopoietic precursor cells (CD34+ progenitors) [61], we first checked the yield and tracked the CD14+ marker expression of M-CSF-stimulated CD14+- and CD34+-derived MΦs along culturing weeks (harvest rounds) from the progenitors producing organoids (Figure 1B). Across harvest rounds, CD14+- and CD34+-derived MΦs displayed a similar phenotype, as shown by brightfield microscopy (Figure 1B). We achieved similar CD14 expression of CD14+- and CD34+-derived MΦs differentiated from 5 × 10^6^ progenitors per T75 flask across harvest rounds (Figure 1B). However, the yield of produced progenitors varied across harvest rounds between the used protocols (Figure 1B). Across sequential harvests, the amount of produced CD14+ progenitors decreased by 1.7-fold. In contrast, the yield of CD34+ progenitors increased across harvest rounds to 2.7-fold (Figure 1B). In total, we were able to produce 68.5 × 10^6^ million CD14+ progenitors and 27 × 10^6^ CD34+ progenitors from 1500 seeded 201B7 cells in three harvest rounds (250 cells per well in a six-well plate). This reflects a 3-fold increase in CD14+ progenitor numbers as compared with the Cui et al. protocol [18]. Furthermore, our CD34+ progenitor production is in alignment with the investigated protocol by Bitzer et al. [19].

One advantage of the iPSC technology is the production of a potentially indefinite numbers of cells for high-throughput screening or drug candidate profiling purposes [21]. To further enhance versatility, we compared the freezing–thawing process of CD14+ and CD34+ progenitors of both differentiation protocols. Upon thawing, the CD34+ progenitors showed high recovery rates (>90% of viability), while this was not achieved with the CD14+ progenitors. Therefore, we generated large frozen stocks of CD34+ progenitors, which after thawing could be differentiated to MΦs in only one week of M-CSF stimulation (Figure 1C). We observed that the CD14 expression was at a similar level to freshly produced CD34+ progenitors (Figure 1C and Figure S1B) and that the cells still actively proliferated during the final maturation step towards MΦs. We concluded that additional enhancement of the MΦ production can be achieved by transferring remaining progenitors for an additional 7 days in culture for an additional three harvest rounds (Figure 1C), which increased the yield of CD34+-derived MΦs by up to 3-fold in alignment with Bitzer et al. [19]. In addition, MΦs generated from frozen CD34+ progenitors showed no obvious difference in surface marker expression (lymphocyte marker CD45, monocytic lineage marker CD14, hematopoietic stem cell marker CD34, MΦ markers CD206, CD163, and CD80) compared with MΦs differentiated from isolated and purified blood monocytes from frozen PBMCs from several donors (Figure S1C), consistent with the published IDM protocol [19].

In summary, the described upscaling and freezing–thawing steps speed up the production of a high-yield and viable IDM stock in a one-step procedure applicable to high-throughput screenings or drug candidate profiling. This aspect and the herein described characterization of the tissue-resident-like IDMs via the monocyte- (CD14+ progenitors) or the hematopoietic stem cell-like (CD34+ progenitors) route highlight that IDMs are suitable as a surrogate compared with frozen PBMC-isolated and purified monocyte-derived MΦs (MDMs), especially for screening purposes when large batches of MΦs without donor-to-donor variations are wanted. Further studies herein will focus on CD34+-derived MΦs, referred to as IDMs. Used MDMs generated from several donors are indicated by name.

### 2.2. The Compound-Induced Polarization of IDMs and MDMs Is Indicated by Cell Shape Changes

**Figure 2:**
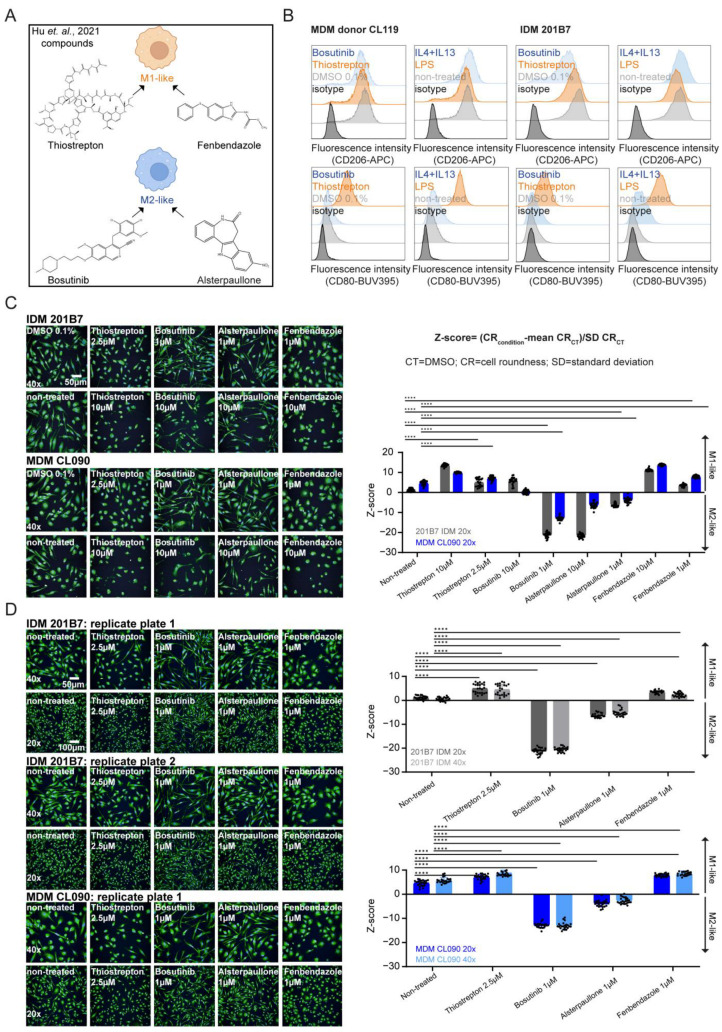
Polarizing compound-induced modulation of MΦ morphology. (**A**) Schematic of Hu et al. 2021 [30], who published M1- and M2-like cpds (created by BioRender.com and ChemDraw 23.1). (**B**) Flow cytometry analysis of CD80 and CD206 expression of cpd-treated (thiostrepton 2.5 μM and bosutinib 1 μM) and biologically stimulated (LPS and IL4+IL13) MΦs (CD34+-derived IDMs versus MDM donor CL119) (in %; *n* = 3; gating is based on a 1% isotype control). (**C**) Equation and Z-score calculation for cell roundness analysis; data are depicted as the mean ± SD from 20× magnification. Representative high-content Opera Phenix^TM^ confocal images (40×; nucleus–Hoechst3342; cytoplasm/ER concanavalin A-Alexa488; one field out of ten) of PhenoVue Kit-stained IDMs and the MDM donor CL090 treated with illustrated conditions for 24 h. Each condition (12 wells; 5 fields per well) was imaged in two replicate 384-well plates. Statistics: two-way ANOVA with significance **** *p* < 0.0001. (**D**) Representative high-content Opera Phenix^TM^ confocal images (40× one field out of ten or 20× one field out of five; nucleus–Hoechst3342; cytoplasm/ER concanavalin A-Alexa488) of PhenoVue Kit-stained IDMs and the MDM donor CL090 treated with illustrated conditions for 24 h. Each condition (12 wells) was imaged in two replicate 384-well plates. Statistics: two-way ANOVA with significance **** *p* < 0.0001. Z-score calculation for cell roundness analysis: data are depicted as the mean ± SD at 20× or 40× magnification.

Previous reports highlighted that pro-inflammatory and anti-inflammatory human and mouse MΦs have distinct morphologies manifested in different cell shapes, indicating their polarization state [30,31,32]. Therefore, cell shape was suggested as a novel screening read-out to identify MΦ-polarizing cpds [30]. Amongst others, two structurally and functionally distinct cpds, namely thiostrepton (a thiopeptide antibiotic and FOXM1 inhibitor) and bosutinib (an Src/Abl tyrosine kinase inhibitor), were identified in a respective phenotypic cpd screen (2086 bioactive cpds, 760 FDA-approved drugs, and 1280 natural cpds) using MDMs pooled from different donors [30]. The authors further validated the potential for MΦ repolarization by these two cpds on the transcriptional level and demonstrated the reprogramming of M2-like tumor-associated MΦs (TAMs) by thiostrepton in vivo, leading to pronounced anti-tumor activity [30]. Based on their thorough functional characterization, we decided to use these cpds as references in our study (Figure 2A). To initially verify MΦ activation induced by bosutinib or thiostrepton treatment, we tracked CD80 (M1 marker) and CD206 (M2 marker) expression by flow cytometry (Figure 2B). Consistently, MDMs and IDMs exhibited an increase in CD80 expression when stimulated with thiostrepton or LPS, with the latter serving as an internal biological control. CD206 marker expression changed slightly upon bosutinib treatment or biological stimulation with IL4+IL13. We explain this phenomenon by the initial high CD206 values seen herein and published elsewhere [19,25] in unstimulated IDMs and MDMs, as verified across several donors (Figure S1C).

To further substantiate the versatility of IDMs in phenotypic screens for the identification of MΦ-polarizing cpds, we assessed cell shape changes in IDMs compared with MDMs by performing confocal high-content imaging of dual-colored MΦs after treatment with reference cpds. In detail, we modified the algorithm described by Hu et al. [30] to first detect a MΦ by its nucleus stain (Hoechst33352 channel) and defined the cell surrounding by the cytoplasm/ER concanavalin A stain (Alexa488 channel). Normalized to the vehicle control (DMSO), the calculated Z-score was determined by cell roundness quantified changes ranging from more rounded (>0) towards more elongated shapes (<0) (Figure 2C). In alignment with the MDM-based findings from Hu et al. [30], we saw a consistent rounded M1-like phenotypic change upon thiostrepton (2.5 μM) and an elongated M2-like phenotypic change upon bosutinib (1 μM) treatment in both MΦ systems, with a high significance of *p* < 0.0001 after 24 h of stimulation (Figure 2C). This observation was further strengthened by the additionally published and thoroughly characterized [30] M2-like cpd alsterpaullone (CDK inhibitor; 1 μM; Figure 2A) and the additional M1-like cpd fenbendazole (microtubule destabilizing agent; 1 μM; Figure 2A) that showed significant round or elongated morphological changes in IDMs and MDMs, respectively (Figure 2C). By visual inspection and Z-score quantification, the range of shapes from round to elongated varied depending on the cpd stimulation and the MΦ system (Figure 2C). The Z-score trend for M1- or M2-like cpd-treated IDMs and MDMs was the same, even if the respective values were not identical for both MΦ systems. This phenomenon can be explained by the different seeding cell density because IDMs and MDMs differ in size and are thereby normalized to their respective vehicle control (DMSO). We also noted that the dose-dependent cell toxicity effects of the four cpds induced a shift in shape towards higher positive Z-scores, thereby erroneously mimicking round cells. As an example, the change of a negative to a positive Z-score by bosutinib treatment (1 to 10 μM) is indicated in Figure 2C.

Considering IDMs as an applicable drug screening and profiling surrogate to MDMs, we performed the experiments in a 384-well format in a semi-automated process (see Materials and Methods 4.6). Due to the size difference of MDMs and IDMs, we also checked if the magnification of our imaged cells (20×: 5 fields per well versus 40×: 10 fields per well) influenced the outcome of our Z-score quantification. The results highlighted in Figure 2D confirmed that Z-score calculation derived from images taken at 20× and 40× magnifications leads to the same outcome in IDMs and MDMs enabling a cell roundness read-out already with a small number of imaged cells or lower resolution capacity.

Taken together, the cell roundness analysis by Z-score calculation is a valuable phenotypic read-out to quantify morphological changes by cpd-induced M1- or M2-like polarization in IDMs and MDMs applicable at 20× and 40× magnification for cpd screening and profiling purposes.

### 2.3. Heterogeneity of Morphological Changes Can Occur upon Compound Treatment and Biological Stimulation of MΦ Polarization

**Figure 3:**
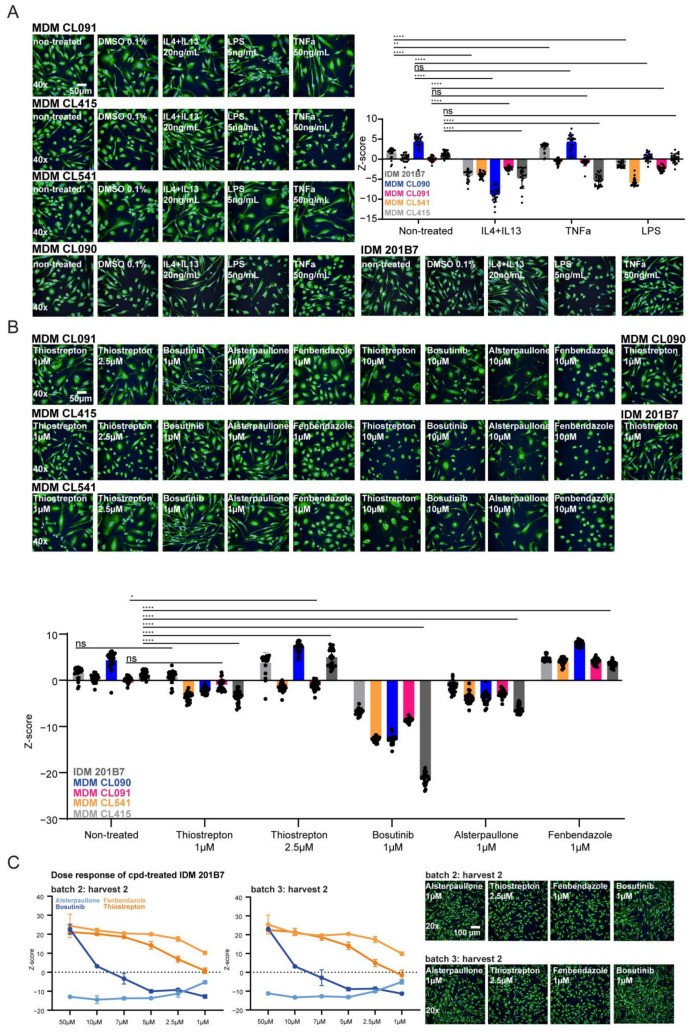
Heterogeneity of morphology changes upon compound treatment and biological stimuli. (**A**) Z-score calculation for cell roundness analysis: data are depicted as the mean ± SD at 20× magnification. Representative high-content Opera Phenix^TM^ confocal images (40×; nucleus–Hoechst3342; cytoplasm/ER concanavalin A-Alexa488; one field out of five) of PhenoVue Kit-stained IDMs and several MDM donors (CL090, CL415, CL541, and CL091) treated with illustrated conditions for 24 h. Each condition (12 wells; 5 fields per well) was imaged in two replicate 384-well plates. Statistics: two-way ANOVA with significance **** *p* < 0.0001 and ** to *p* < 0.001. ns = non-significant. (**B**) Conditions as in A with described cpd stimulation. Statistics: two-way ANOVA with significance **** *p* < 0.0001 and * to *p* < 0.01. ns = non-significant. (**C**) Dose–Response analysis of Z-score calculated cell roundness. Data are depicted as the mean ± SD. Representative high-content Opera Phenix^TM^ confocal images (20×; nucleus–Hoechst3342; cytoplasm/ER concanavalin-Alexa488; one field out of five) of IDMs treated with illustrated conditions (alsterpaullone, bosutinib, thiostrepton, and fenbendazole) for 24 h. Each condition (4 wells; 5 fields per well) was imaged in one 384-well plate. IDMs were generated from different frozen CD34+-progenitor stocks (harvest) across different progenitor production rounds (batch).

Transcriptome profiling of the four reference cpds (bosutinib, thiostrepton, fenbendazole, and alsterpaullone [30]) in pooled MDM donors has demonstrated that many new pathways distinct from the biological LPS and IL4+IL13 stimulation were induced, revealing a remarkable plasticity of MΦ activation [30]. To unravel the spectrum of MΦ polarization, we addressed cell shape changes induced by cpd treatment compared with biological stimulation in both MΦ systems (Figure 3A,B). Regarding biological stimulation, we observed a significant elongated effect upon IL4+IL13 stimulation in IDMs as well as in MDMs (Figure 3A). However, we faced no significant strong round effect upon LPS stimulation in the Z-score calculation, even if flow cytometry data confirmed high CD80 expression levels upon LPS treatment at 24 h to 48 h (Figure 2B and Figure S2A). This result does not align with the findings published by Hu et al. [30] in MDMs, which might be explained by the difference between IDMs and MDMs. Therefore, we checked the phenotypic changes in several different donors and included TNFα as another pro-inflammatory stimulus in our study. Again, we detected variance in response across donors, resulting in minimal non-significant changes for LPS and TNFα treatments in the roundness score (Figure 3A). We argue that the cell culture medium composition of RPMI-1640 used in Hu et al. [30] in contrast to the DMEM used in our study drives these differences. It is known that non-essential amino acids (asparagine, aspartic acid, glutamic acid, and proline) impact the phenotype of human MDMs that was supplemented in our study [62,63]. The most abundant amino acid in human glutamine, or the more stable synthesized version L-alanyl-glutamine (GlutaMAX), was the most prominent component that varied in the cell culture medium composition. The impact of glutamine metabolism on metabolic reprogramming in polarized MΦs for bioenergetic and biosynthetic demand was highlighted in several studies [64,65,66,67].

Regarding stimulation with cpds, we saw similar changes in cell shape at the same concentrations for IDMs and MDMs (Figure 2C and Figure 3B). However, thiostrepton induced a somewhat heterogeneous shape change across MDMs from different donors at different concentrations (1 μM and 2.5 μM), although these MDMs had similar expression of surface markers. We argue that the pooling of donors [30] might level out individual Z-score variations (Figure 3B and Figure S1C).

Based on these observed donor variations, we questioned if the harvest rounds of CD34+ progenitors and batches of generated IDMs would affect the Z-score calculation as well. Strikingly, we verified in a dose–response assay consistent morphological effects independent of harvest and batch usage (Figure 3C and Figure S2B). This aspect emphasizes the IDMs as an appropriate cell model to unravel cpd-induced morphological changes for drug screening or profiling purposes (Figure 3C and Figure S2B).

In conclusion, using the Z-score calculation for cell roundness, the heterogeneity, and thereby the spectrum of phenotypes across biological and cpd stimuli in a time- and dose-dependent manner revealed that they vary primarily in several donors of MDMs. Therefore, we aimed to unravel if cell roundness was the only determining morphological parameter to indicate MΦ polarization by phenotypic profiling using the cell painting assay.

### 2.4. Cell Painting Features Describe the Phenotypic Spectrum of MΦ Polarization upon Compound Treatment and Biological Stimulation

**Figure 4:**
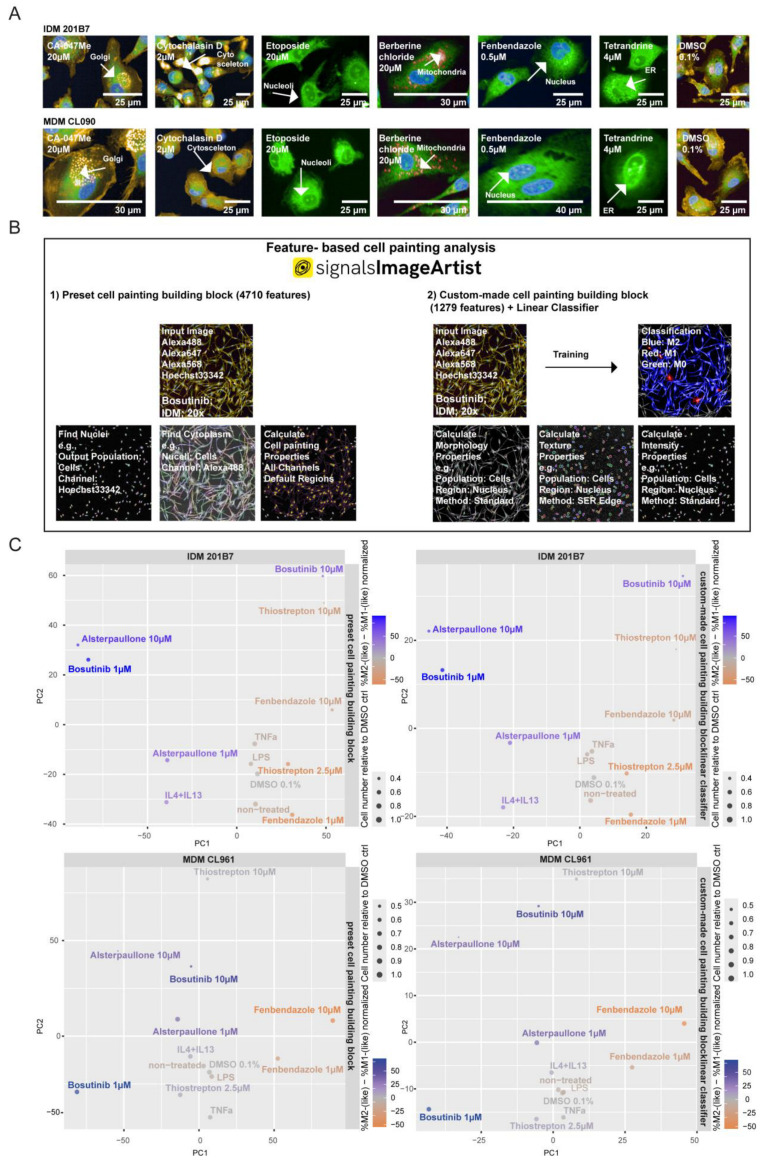
Cell painting features for the description of the phenotypic spectrum of MΦ polarization. (**A**) Representative high-content Opera Phenix^TM^ confocal images of cell-painted (PhenoVue Kit: PhenoVue 641 mitochondrial stain, PhenoVue Hoechst 33342 nuclear stain, PhenoVue Fluor 488-concanavalin A, PhenoVue 512 nucleic acid stain, PhenoVue Fluor 555-WGA and PhenoVue Fluor 568-Phalloidin) IDMs and an MDM donor CL090 with indicated cell painting controls (berberine chloride, fenbendazole, etoposide, cytochalasin D, CA-074Me and tetrandrine; 12 wells per condition; 10 fields per well; from two independent replicate 384-well plates) after 24 h of stimulation. The effect of the cpds on the respective cellular compartment and organelles is indicated by the white arrows. (**B**) Schematic of the established feature-based analysis using SImA: ‘preset cell painting building block’ and ‘custom-made cell painting building block combined with linear classifier’. bosutinib-treated IDM Opera Phenix^TM^ confocal imagery is shown as a representative example. The whole analysis pipeline is described in Materials and Methods 4.8. (**C**) Principal component analysis of ‘Preset cell painting building block’ and ’Custom-made cell painting building block’ features indicated cpd- and biologically stimulated IDMs and an MDM donor CL961 for 24 h. Each datapoint indicates the mean of 4 replicate wells (5 fields per well; 20× magnification) per condition. ‘Preset cell painting building block’ is based on 4710 used features. ’Custom-made cell painting building block’ is based on 1279 features.

The cell painting technique has been rapidly adopted for phenotypic drug discovery, combining cell and computational biology to describe the status of stimulated cells with thousands of features generated during image and data analysis [46,51,55,68,69,70,71,72]. Therefore, we reasoned if cell painting could be highly suitable to describe MΦ-polarizing cpds using feature extraction and phenotypical profiling. Consequently, we established the cell painting of MΦs by labeling them with the recommended dyes (PhenoVue Fluor 555-WGA, PhenoVue Fluor 568-Phalloidin, PhenoVue 512 nucleic acid stain, PhenoVue Hoechst 33342 nuclear stain, PhenoVue Fluor 488-concanavalin A and PhenoVue 641-mitochondrial stain) [33,34]. To generate a common cell painting protocol suitable for both IDMs and MDMs, we optimized the published cell painting protocol, adjusting the instrumentation and labeling conditions. Thereby, we titrated the labelling dyes to minimize spectral overlap and to set up the assay for both MΦ systems at 20× and 40× magnification (see Materials and Methods 4.6). In addition, we used the published control cpds (tetrandrine, fenbendazole, etoposide, cytochalasin D, CA-047Me, and berberine chloride) that affect the cellular compartments stained by the individual cell painting dyes [33,35,73,74]. In Figure 4A, the expected giant multinucleated cells by fenbendazole, the abundant ER by tetrandrine, the large nucleoli by etoposide, the modulated Golgi abundance by CA-074Me, the redistribution of mitochondria by berberine chloride, and the disrupted actin cytoskeleton by cytochalasin D treatment could be detected in both MΦ systems.

For image and data analysis, we used the Signals Image Artist software (SImA1.3) that includes a building block for the analysis of cell painting assays. First, we decided to process the recommended cell painting data analysis pipeline calculating the intensity, the morphology and the texture properties from each channel across defined regions (see Materials and Methods 4.8; Figure 4B; ‘preset cell painting building block’). In total, the ‘preset cell painting building block’ allows for the extraction of 4710 different features. However, a classification of subpopulations like M0, M1(-like) and M2(-like) cannot be combined with the ‘preset cell painting building block’ in the PhenoLOGIC™ technology [75] included in SImA. Therefore, we manually set up an analysis pipeline of building blocks calculating 1279 cell painting features based on intensity, morphology, and texture properties (Figure 4B; ‘custom-made cell painting building block’).

First, we performed a principal component analysis (PCA) to compare the MΦ polarization projections generated by the ‘custom-made cell painting building block’ versus the ‘preset cell painting building block’. We observed that the intrinsic patterns of M1(-like) versus M2(-like) stimulation were similar in both analysis pipelines for 201B7 IDMs and MDM donor CL961 (Figure 4C). This finding indicated that the reduced number of features present in the ‘custom-made cell painting building block’ is already sufficient to describe MΦ polarization patterns. Moreover, we detected that the distribution of patterns was more similar within IDMs (201B7 and ChiPSC12 line) in contrast to MDMs (donor CL414 and CL961) systems (Figure S3A and Figure 4C). In addition, different treatment groups (biologicals versus cpds) clearly separated from each other in the PCA, highlighting the multidimensional plasticity of MΦs beyond the traditional M1/M2 paradigm manifested by biological stimulation. Within the groups of M1- and M2-like cpds, distinct patterns were observed as well, which emphasize the potential of cell painting to discriminate different MoA and pathways to induce the polarization of MΦs. In addition, cell toxicity-inducing conditions (e.g., bosutinib 10 μM and thiostrepton 10 μM) are readily identified by the decreased cell number in the PCA plot (Figure S3A and Figure 4C). Of note, biological 384-well replicates clustered tightly for each condition, indicating the reproducibility of the system (Figure S3B).

In summary, cell painting feature profiling enables the description of MΦ polarization combined with the parallel detection of cytotoxic-inducing conditions. Therefore, the evaluation of cell painting feature fingerprints is a novel tool for deciphering the phenotypic spectrum of MΦ polarization modulated by biologicals and cpds. The comparison between the 4710-feature set from the SImA ‘preset cell painting building block’ to the ‘custom-made cell painting building block’ highlights that 1279 features are already sufficient to separate different M1- and M2(-like) conditions with a reduced feature set.

### 2.5. Compound-Induced MΦ Polarization Effects Are Identified and Quantified by Feature-Based Cell Painting Analysis

Next, we extended the analysis pipeline for not only discriminating M1- and M2(-like) conditions, but also to quantify the modulation into pro- or anti-inflammatory states. Therefore, we combined the ‘custom-made cell painting building block’ (1279 features) with the ‘linear classifier’ method from the PhenoLOGIC™ technology [75] included in SImA (Figure 4B). This ‘linear classifier’ allows for the classification of subpopulations, which we trained on the M0, M1(-like), and M2(-like) states by selecting an internal control set. To that end, we randomly selected 100 cells per population across different wells, fields, replicates, and 384-well plates, which were used to train individual conditions (see Materials and Methods 4.8). Due to the distinct pattern describing biological versus cpd treatment illustrated in the PCAs (Figure 4C and Figure S3), we considered training models based on biological stimulation, cpd stimulation, or a combination of both. Moreover, we used IDMs for the training based on their great robustness and homogeneity, as demonstrated within this study (Figure 2D, Figure 3C and Figure 4C).

**Figure 5:**
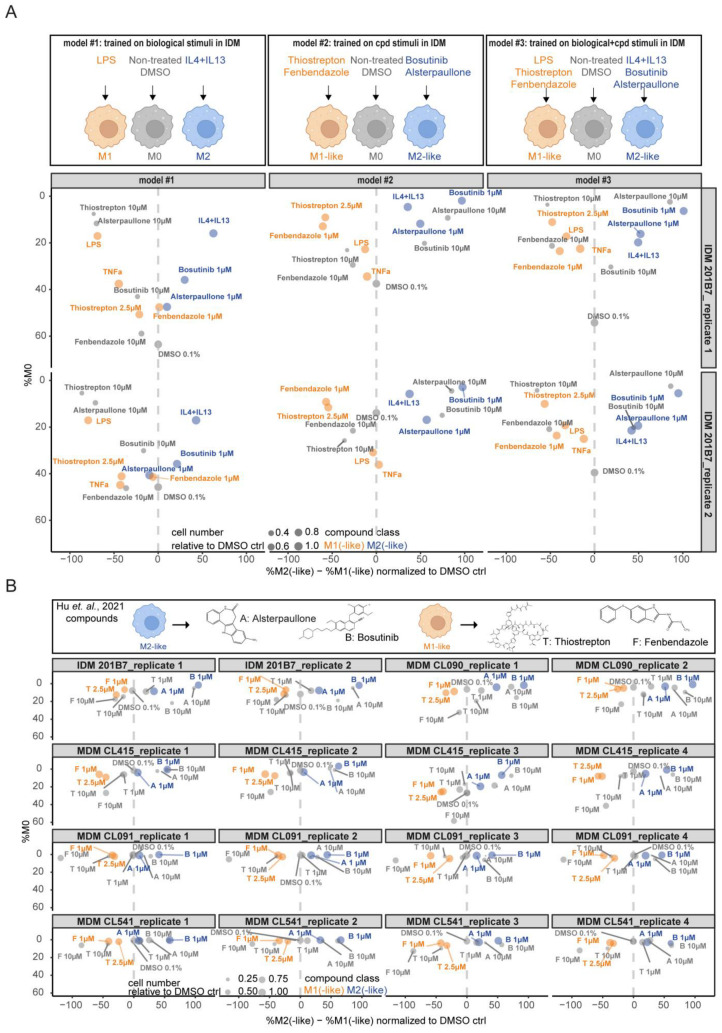
‘Linear classifier’ analysis for the quantification of M1 and M2(-like) compound states. (**A**) Schematic of SImA ‘linear classifier’ IDM-trained models 1-3 (created by BioRender.com). Quantification of the indicated 24 h of cpd- and biologically stimulated IDMs from two 384-replicate plates. DMSO 0.1%-normalized values %M2(-like)—%M1(-like) correspond to a mean of 4 wells imaged at 20× magnification (replicate 1 and 2; 5 fields per well). DMSO-normalized %M2(-like)—%M1(-like) values are listed in Table S1. (**B**) SImA ‘linear classifier’ analysis of the indicated 24 h of cpd-stimulated IDMs and several MDM donors (CL090, CL091, CL541, and CL415) from two to four 384-well replicate plates by model 2 from A trained on IDMs. DMSO-normalized %M2(-like)—%M1(-like) values correspond to a mean of 12 wells imaged at 20× magnification (5 fields per well). DMSO-normalized %M2(-like)—%M1(-like) values are listed in Table S2. The four used reference cpds from [30] are indicated abbreviated in the figure.

In the first model (model 1), only biological stimuli (LPS and IL4 + IL13), non-treated IDMs, and the vehicle control (DMSO) were used for training (Figure 5A). To train the second model (model 2), cpd stimuli inducing M1-like (thiostrepton 2.5 μM and fenbendazole 1 μM), M2-like (bosutinib 1 μM and alsterpaullone 1 μM), and M0 (non-treated and DMSO) were used. We decided to use all four cpds to enable the identification of a spectrum of several MoAs and MΦ activation states. In the third model (model 3), we combined biological and cpd stimulation.

In Figure 5A, the outcome of models 1–3 are illustrated in IDMs showing consistent results across the two replicate 384 well-plates (replicate 1 and 2). Cell toxicity-inducing compound concentrations are indicated by the smaller circles containing the information about the cell number. The strongest polarizing effects represented by the shift on the y-axis were detected with models 2 and 3. Comparing the DMSO-normalized %M2(-like)—%M1(-like) values, we did not see drastic differences using model 2 or model 3 in contrast to model 1 (Table S1). Importantly, model 2 enabled the identification of biological stimuli, although these conditions were not trained in this model (Figure 5A). Considering that biological stimuli such as LPS could be detected with model 2, even if the cell roundness analysis did not provide a strong Z-score (Figure 3A), we decided to further progress with model 2.

To design one ‘linear classifier’ as an analysis tool applicable for IDMs and MDMs, we applied the IDM-trained model 2 for cpd and biological stimulation on images from several different donors (Figure 5B), which were already evaluated in here by surface marker expression and cell roundness analysis (Figure 2, Figure 3, Figure 4 and Figure 5). This step was highly relevant to determine, quantify, and compare MΦ-polarizing cpd effects within both MΦ systems—a benefit over the cell roundness analysis. Strikingly, across several replicate 384-well plates, we found that our model 2 is suitable to classify M1- and M2-like cpds also in several MDM donors (Figure 5B). Most importantly, thiostrepton that did not induce a significant rounded phenotype by the cell roundness analysis in donors CL091 and CL541 was clearly identified as M1-like stimulus by the ‘linear classifier’ analysis (Figure 5B versus Figure 3B; Table S2). Moreover, a hierarchy of the best responding donor can be determined by comparing the DMSO-normalized %M2(-like)—%M1(-like) values for each condition (Table S2).

Taken together, the ‘linear classifier’ model 2 benefits over the cell roundness analysis because it enables the identification of MΦ-polarizing cpds and the quantification of their effect size in MDMs as well as in IDMs.

### 2.6. Compound-Induced MΦ Reprogramming Effects Are Identified and Quantified by Feature-Based Cell Painting Analysis

In many therapeutic settings, the reprogramming of polarized MΦs, e.g., repolarization of M2-like TAMs towards M1-like MΦs with anti-tumor capabilities, is warranted [76,77,78]. Therefore, we tested whether our ‘linear classifier’ is also capable of identifying cpds with repolarization potential and quantifying their effects.

**Figure 6:**
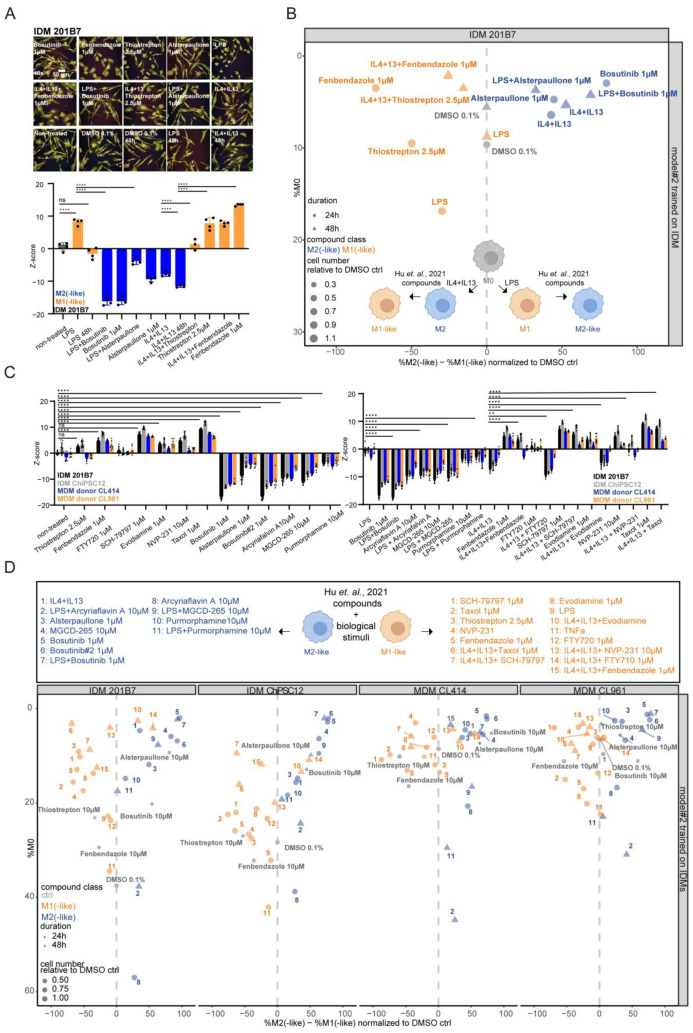
Identification and quantification of compound-induced MΦ reprogramming effects. (**A**) Representative high-content Opera Phenix^TM^ confocal images of cell-painted (PhenoVue Kit: PhenoVue 641 mitochondrial stain, PhenoVue Hoechst 33342 nuclear stain, PhenoVue Fluor 488-concanavalin A, PhenoVue 512 nucleic acid stain, PhenoVue Fluor 555-WGA, and PhenoVue Fluor 568-Phalloidin) IDMs with indicated M1- and M2-like cpds single-treated or combined with biological stimuli (4 wells per condition; 5 fields per well; 20× magnification) for 24 h or 48 h. Z-score calculation for cell roundness analysis: data are depicted as the mean ± SD at 20× magnification (from one 384-well plate). Statistics: two-way ANOVA with significance **** *p* < 0.0001. ns = non-significant. (**B**) SImA ‘linear classifier’ quantification of IDMs trained on IDM model 2 from Figure 5A. DMSO-normalized %M2(-like)—%M1(-like) values correspond to a mean of 4 wells imaged at 20× magnification (5 fields per well). DMSO-normalized %M2(-like)—%M1(-like) values are listed in Table S3. Treatment conditions used from A. Normalization was performed for the duration of the treatment. (**C**) Z-score calculation for cell roundness analysis of cell-painted (PhenoVue Kit: PhenoVue 641 mitochondrial stain, PhenoVue Hoechst 33342 nuclear stain, PhenoVue Fluor 488-concanavalin A, PhenoVue 512 nucleic acid stain, PhenoVue Fluor 555-WGA, and PhenoVue Fluor 568-Phalloidin) IDMs (ChiPSC12 and 201B7) and two MDM donors (CL961 and CL414) with indicated M1- and M2-like cpds, single-treated or combined with biological stimuli (4 wells per condition; 5 fields per well; 20× magnification) for 24 h or 48 h. Data are depicted as the mean ± SD at 20× magnification (from two independent 384-well plates). Statistics: two-way ANOVA with significance **** *p* < 0.0001 and ** *p* < 0.01. ns = non-significant. (**D**) SImA ‘linear classifier’ analysis of indicated 24 h cpd- and biologically stimulated IDMs (201B7 and ChiPSC12) and two MDM donors (CL414 and CL961) from one 384-well plate trained on IDM model 2 from Figure 5A. DMSO-normalized %M2(-like)—%M1(-like)values correspond to a mean of 4 wells imaged at 20× magnification (5 fields per well). DMSO-normalized %M2(-like)—%M1(-like)values are listed in Table S4.

Experimentally, MΦ reprogramming was induced by 24 h of stimulation with LPS or IL4 + IL13 followed by the 24 h cpd treatment with the initial stimuli still present (no wash-out), as recommended by [30]. First, we tracked the morphological changes in a time course live cell image analysis in IDMs, indicating MΦ reprogramming induced by cpd stimulation (Figure S4A). This finding underlines that IDMs can be reprogrammed in the same manner as MDMs, as described in [30]. Next, we performed an LDH assay to prove that no cell toxicity effects are happening across the described reprogramming procedure in our IDM system (Figure S4B). While 24 h of LPS treatment induced a round phenotype, subsequent treatment with bosutinib or alsterpaullone for 24 h in the presence of LPS led to a more elongated phenotype (Figure 6A). Similarly, thiostrepton and fenbendazole were confirmed to reprogram IDMs that were initially polarized by IL4 + IL13 (Figure 6A). Therefore, the results of the Z-score analysis at 24 h and 48 h corroborated the shape changes seen already in the time course analysis in Figure S4A. Using the reference cpds for repolarization, the ‘linear classifier’ analysis was tested on the described dataset to quantify reprogramming effects (Figure 6B; Table S3). Strikingly, for all four cpds (bosutinib, thiostrepton, alsterpaullone, and fenbendazole), the reprogramming could be quantified with our model 2. The effect sizes of repolarization differed quantitatively from those achieved by the polarizing cpds alone, potentially due to the persisting presence of the initial biological stimulus. Importantly, the outcome was not impacted by normalizing the data to DMSO at 24 h or 48 h (Figure S4C; Table S3).

Next, we aimed to evaluate our generated ‘linear classifier’ model 2 by testing a larger cpd set of published and functionally characterized MΦ-polarizing cpds [30]. These additional cpds include a broader spectrum of chemical classes with different MoAs: SCH-79797, evodiamine, MGCD-265, arcyriaflavin A, purmorphamine, NVP-231, FTY720, and taxol. To illustrate the robustness of our experimental setup, we included our M2-control bosutinib from different stocks across the 384 well-plates (named bosutinib and bosutinib#2).

As mentioned by Hu et al. [30], the additional M1- or M2-like cpds induced respective consistent concentration-dependent shape changes in MDMs from several donors (donor CL414 and donor CL961) as well as in IDMs generated from two different cell lines (201B7 and ChiPSC12 line) (Figure 6C and Figure S4D). These concentration-dependent shape changing effects were quantified using the ‘linear classifier’ (Figure S4E). As an example, the drastic shape change towards a more rounded phenotype induced by NVP-231 from 1 μM to 10 μM can be quantified in the DMSO-normalized %M2(-like)—%M1(-like)values for both MΦ systems (Figure S4D,E; Table S4).

Next, we addressed the reprogramming capacity of the M1- or M2-like cpds using their effective concentration to induce MΦ polarization, indicated by cell shape and confirmed by the ‘linear classifier’ analysis. Interestingly, not all tested MΦ-polarizing cpds had the ability to induce a reprogramming phenotype indicated by the reversed Z-score (Figure 6C and Figure S4F). In detail, M1-like cpds SCH-79797 and taxol were able to reverse the shape changes when pre-treated with IL4 + IL13 across the MΦ systems (Figure 6C and Figure S4F). In line with this, all tested M2-like cpds were able to reprogram the MΦs when combined with LPS stimulation (Figure 6C and Figure S4F). This finding was confirmed with the DMSO-normalized %M2(-like)—%M1(-like) values determined by the ‘linear classifier’ (Figure 6D). In this context, the combination of cell roundness and ‘linear classifier’ analyses also informs us about donor-specific repolarization effects (e.g., IL4 + IL13 + evodiamine in MDM donor 961 and IL4 + IL13 + NVP-231 in MDM donor 414) (Figure 6C,D). Moreover, as indicated by the range of the Z-score, the classification of the strongest polarization effects within the specific modulator class could be determined in the ‘linear classifier’ analysis across the MΦ systems (Table S4, Figure 6D). Because this classification refers to the 201B7 IDMs, we decided to generate a model trained on MDM donor CL414 and compare its classification with the classification obtained by the IDM-trained model. Importantly, we found that the normalized %M2(-like)—%M1(-like) values change; however, the trend of the polarization effects are similar (Figure S4E; Table S5).

In conclusion, the herein developed IDM ‘linear classifier’ model 2 is suitable not only for identifying and quantifying MΦ polarizing but also reprogramming changes induced by cpds across both MΦ systems. This finding is underlined by the testing of a larger spectrum of structural and functionally distinct published MΦ (re-)polarizing cpds.

### 2.7. Cell Roundness Is Not Scored as the Relevant Morphological Feature to Discriminate Compound-Induced M1(-like) from M2(-like) Polarization

**Figure 7:**
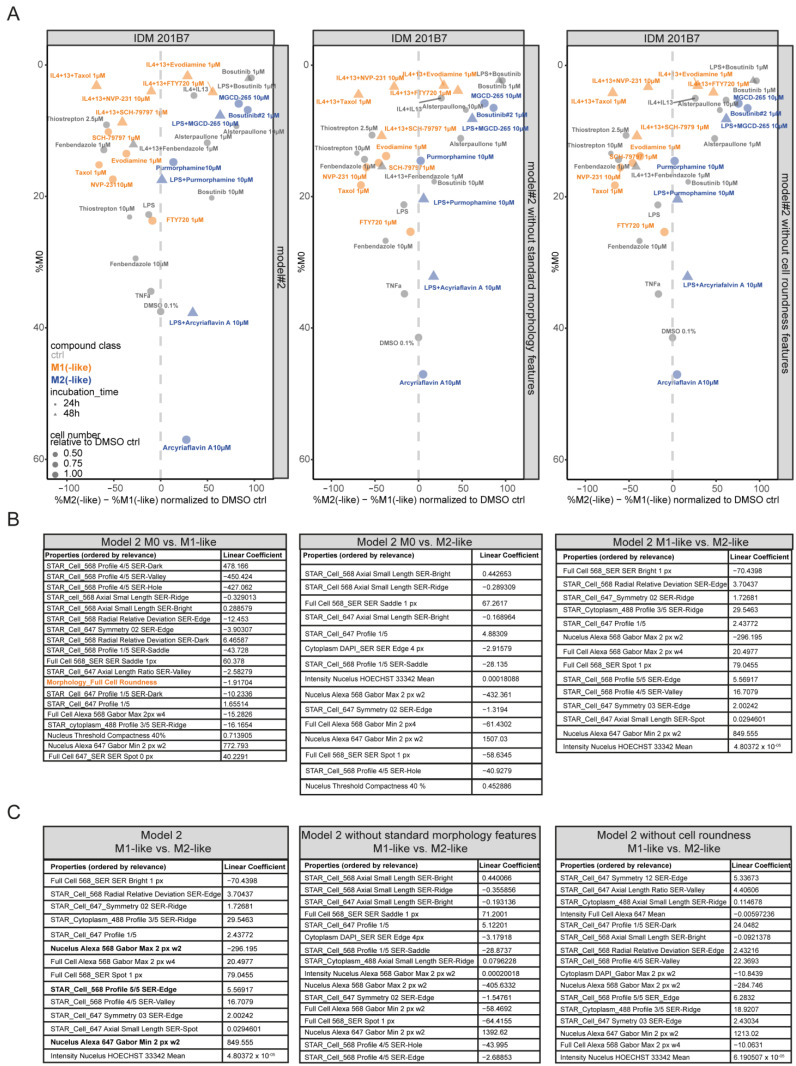
Relevance of standard morphological properties and cell roundness feature for compound-induced MΦ (re-)polarization. (**A**) SImA ‘linear classifier’ analysis of indicated 24 h treated cpd- and biologically stimulated IDMs from one 384-well plate trained on IDM model 2 from Figure 5A. Model 2 differs in the usage of all or no standard morphology (area, roundness, width, length, and ratio of width to length) or no cell roundness features. DMSO-normalized %M2(-like)—%M1(-like) values correspond to a mean of 4 wells imaged at 20× magnification (5 fields per well). DMSO-normalized %M2(-like)—%M1(-like) values are listed in Table S6. (**B**,**C**) List of relevant features for M0, M1-like, and M2-like classification and their corresponding linear coefficient value based on the three models in A. The cell roundness feature is highlighted in orange. The common relevant features of all three ‘linear classifiers’ are illustrated in bold.

Our next aim was to understand what impact and relevance the morphological properties, such as the cell roundness, have for describing MΦ polarization and reprogramming and enabling a discrimination of the cpd-induced M1-like from M2-like effects illustrated in Figure 6D. Consequently, we modified the ‘linear classifier’ by training the IDM model without the cell roundness feature or without the standard morphology features (area, roundness, width, length, and ratio of width to length). Importantly, we observed no drastic changes in the MΦ polarization outcome in all three different ‘linear classifiers’ (Figure 7A; Table S6). Of note, we detected that cytotoxicity conditions (e.g., 10 μM bosutinib or 10 μM thiostrepton) varied in the ‘linear classifier model 2′ towards without standard morphology or cell roundness features (Figure 7A; Table S6). Thus, we compared the list of the relevant features in the three ‘linear classifiers’. Strikingly, we figured out that the cell roundness feature is only scored as relevant when distinguishing unpolarized M0 to M1(-like) (Figure 7B). Importantly, the relevant features to distinguish M1(-like) versus M2(-like) only partially overlap in all three different ‘linear classifiers’, without impacting the cpd classification in the ‘linear classifiers’ without standard morphology or cell roundness features at all (Figure 7C).

In conclusion, the ‘linear classifier’ analysis revealed that the cell roundness feature is not scored as relevant parameter to distinguish M1(-like) or M2(-like) polarized effects in MΦs. Cell roundness plays a determining role to distinguish M0 from M1(-like) states. This initial finding illustrates the impact that morphological feature profiling has in describing the cellular state of MΦ (re-)polarization.

### 2.8. Deep Learning-Based Cell Painting Analysis Confirms Feature-Based Identified MΦ (Re-)Polarization Effects

In phenotypic profiling, DL can be coupled with cell painting approaches to extract biologically meaningful representations from the image-based feature profiles [46,47,48,49,50]. Together with ScreeningHub ValueData, we aimed to establish a DL-based cell painting analysis tool to verify the MΦ (re-)polarization effects identified by the feature-based ‘linear classifier’. In alignment with the ‘linear classifier model 2′, we used a DL algorithm for training 201B7 IDM images that were randomly selected across imaged fields per wells of 384-well plates treated with the conditions of model 2 (see Materials and Methods 4.9; named: DL-model 2). In detail, we extended the training set by incorporating conditions of cell toxicity next to polarization induction, thereby informing about M0, M1-like, M2-like, and dead states (Table S7).

**Figure 8:**
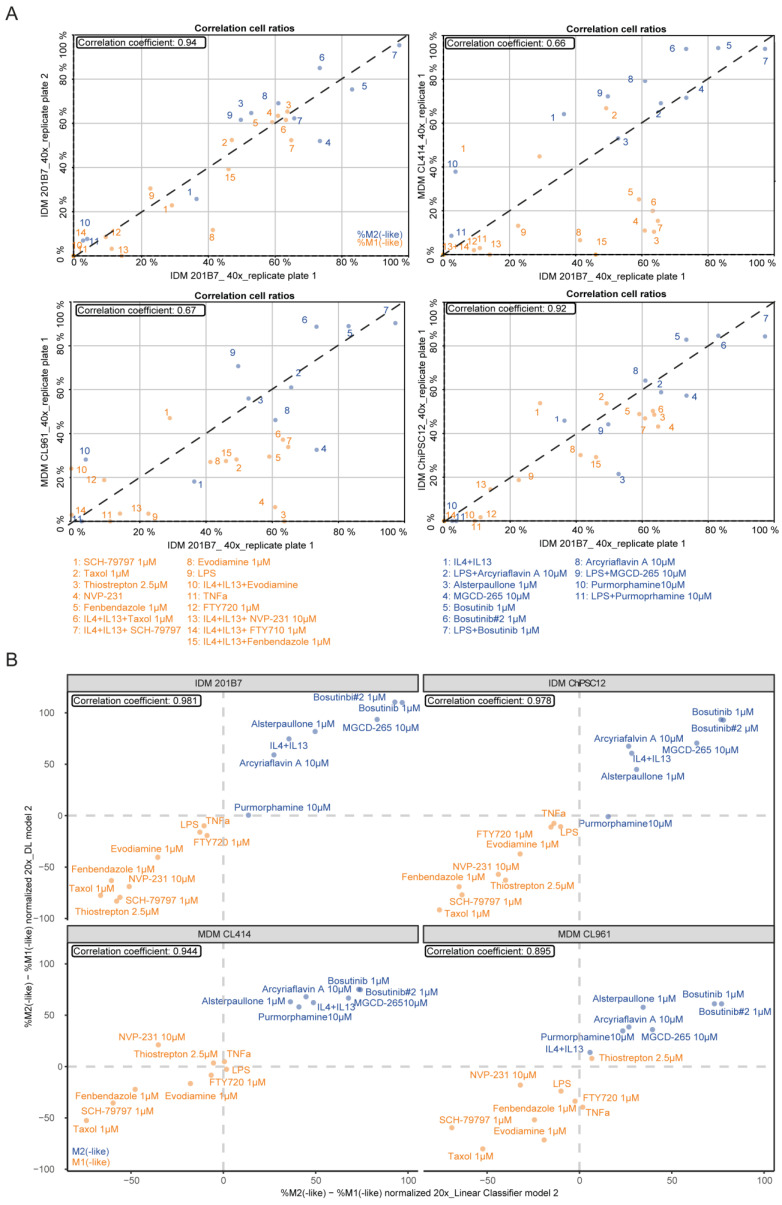
DL-fueled cell painting analyses for MΦ (re-)polarization effects. (**A**) Scatter plot correlating the cell ratios (% M1(-like) and M2(-like) proportion) of the DL-fueled analysis of 24 h cpd- and biologically stimulated IDMs (201B7 and ChIPSC12) and MDMs (CL414 and CL961) from one 384-well plate trained on the IDM DL-model 2 (40× magnification). DMSO 0.1%-normalized values correspond to a mean of 4 wells imaged at 40× magnification (10 fields per well). The correlation coefficient was calculated by the Pearson correlation. (**B**) Scatter plot correlating the cell ratios (DMSO normalized %M2(-like)—%M1(-like); Table S7) of the DL-based and ‘linear classifier’ analyses of conditions in A using 20× magnification (5 fields per well). The correlation coefficient was calculated by the Pearson correlation.

First, we determined if the DL-model 2 describes the MΦ (re-)polarization effects in IDMs (ChiPSC12 and 201B7) as well as MDMs (donors CL414 and CL961). The DL-model 2 identified the same M1- and M2-like cpds as determined by the ‘linear classifier’ model 2. As expected, the highest correlation coefficients were observed for both IDM systems (correlation coefficient > 0.9) but also reached a correlation coefficient >0.6 for both tested MDM donors (Figure 8A). This aspect highlights that the IDM-trained DL-based analysis tool is suitable for identifying and quantifying MΦ (re-)polarization effects across both MΦ systems—a finding that confirms the effects seen with the feature-based ‘linear classifier’ tool.

Because the ‘linear classifier’ model 2 was developed using 20× magnified images for training in contrast to the 40× images used for the DL-model 2, we tested whether both magnifications would be sufficient for a reliable determination of MΦ (re-)polarizing cpds. Thus, we tested the conditions in Figure 8A using either the 20× or the 40× images from two replicate IDM plates. We observed high correlation coefficients of >0.8 values for both magnifications (Figure S5A). This indicates that potent MΦ-polarizing and reprogramming cpds can be found when testing 20× magnified images on a 40× trained DL-model 2, which is highly useful for high-content imaging.

Following, we incorporated the DMSO-normalized %M2(-like)—%M1(-like) values derived from the DL-model 2 and the ‘linear classifier’ model 2, showing that both analysis tools describe the MΦ polarization effects of IDMs (ChiPSC12 and 201B7) and MDMs (donors CL414 and CL961) with correlation scores ranging from 0.89 to 0.98 (Figure 8B; Table S7).

In conclusion, the DL-based analysis of cell painting images confirmed the identification of cpds with MΦ-polarizing capacity and also enabled the quantification of their polarization effects in both MΦ systems. Thus, our established AI-fueled analysis tools are suitable for high-throughput and high-content screening applications.

## 3. Discussion

In the current study, we evaluated and refined methods [18,19] for the in vitro generation of MΦs derived from the iPSC cell line 201B7, because in the past, large-scale phenotypic screens and other in vitro studies on human MΦs had been hampered by the limited availability of suitable cells. While cell lines like THP-1 (a human acute monocytic leukemia cell line) or U937 (a cell line derived from a patient with histiocytic lymphoma) can be used to generate high numbers of uniform macrophage-like cells [25,79], they incompletely recapitulate the biology of tissue-resident MΦs [11,12,13,14,15]. Primary MΦs generated by differentiation from isolated blood monocytes are widely used as in vitro surrogates of tissue-resident MΦs, but their numbers are limited due to the restricted availability of blood donations and the inter-donor variance [16,17]. Herein, we described how frozen stocks of 201B7-derived progenitors can be generated in potentially unlimited numbers and can be thawed with a high recovery rate for subsequent final differentiation towards IDMs in a one-step procedure (Figure 1C). Importantly, IDMs generated from frozen CD34+ progenitors showed no obvious difference regarding surface marker expression compared with CD14+ progenitors and MDMs from several donors differentiated from blood monocytes that were purified from frozen PBMCs (Figure S1C and Figure 1B). Thus, we further refined the generation of highly uniform IDMs that function as a suitable screening surrogate of MDMs applicable in phenotypic screens, as demonstrated by recent studies of our group and others [19,21].

Modulating MΦ polarization is a promising approach with enormous therapeutic potential, because MΦ polarization is critically involved in a plethora of human pathologies [7,8,9,10]. For example, the emerging role of MΦs as critical regulators of tumor immunity led to an interest in targeting MΦs in cancer, and preclinical studies have demonstrated efficacy across therapeutic modalities and tumor types [77,78]. TAMs represent one of the most abundant immune cell types in tumors and mainly originate from peripheral blood monocytes and tissue-resident MΦs [76]. TAMs can promote the initiation and metastasis of tumor cells, inhibit antitumor immune responses, promote resistance against radiotherapy and chemotherapy, and stimulate tumor angiogenesis and, subsequently, tumor progression. A number of strategies have been developed to address the tumor-promoting role of TAMs. These can be broadly divided into two groups: reducing the number of TAMs within the TME or altering their functionality [80,81]. Reducing the recruitment of monocytes into the TME by blocking CCL2-CCR2 signaling or decreasing the differentiation of monocytes into TAMs and reducing their survival in the TME by the inhibition of CSF1-CSF1R signaling showed efficacy as monotherapies in a limited number of tumors in clinical trials [76]. Of note, CSF-1R inhibition induced MΦ repolarization to an anti-tumor phenotype and blocked tumor progression in a mouse glioma model [82]. Based on efficacy in preclinical models, several combination therapies are currently investigated in clinical trials [78]. An intrinsic downside of depleting TAMs is the loss of their latent immune stimulatory role as a primary phagocyte and antigen-presenting cell type within tumors. Therefore, repolarizing TAMs towards an anti-tumor phenotype is regarded as an attractive approach for augmenting other forms of immunotherapy [78]. CD47 is regarded as a ‘do not eat me’ signal overexpressed by tumor cells to avoid being phagocytosed by MΦs. Therefore, blocking the CD47/SIRP-alpha axis can enhance the phagocytic properties of MΦs. In addition to enhanced direct tumor cell killing, anti-CD47 treatments have been shown to repolarize TAMs to an anti-tumor phenotype in preclinical models [81,83]. In preclinical tumor models, intratumoral injection of a TLR2 agonist or targeted delivery of nanoparticles loaded with a TLR7/8 agonist have also been demonstrated to induce repolarization of TAMs towards an anti-tumor phenotype [84,85]. The activating receptor CD40 on the MΦ surface has been targeted with agonistic anti-CD40 antibodies, which mimic the ligand CD40L, expressed by activated T cells. In preclinical studies, anti-CD40 antibodies were effective in repolarizing TAMs into tumor-suppressing MΦs. This preclinical evidence stimulated clinical studies of numerous anti-CD40 agonistic antibodies in combination with checkpoint immunotherapy, chemotherapy, or targeted therapies in patients with advanced solid tumors [86]. These non-exhaustive examples from oncology underscore the potential translational relevance of identifying cpds and targets for the (re-)polarization of MΦs. This is further substantiated by the identification of thiostrepton in a phenotypic screen for MΦ modulators by [30], an antibiotic capable of repolarizing TAMs into a tumor-suppressive MΦ phenotype that exhibited potent anti-tumor activity in mice and was consequently used as a reference cpd in this study [30].

Therefore, better understanding of MΦ plasticity and the identification of MΦ-polarizing cpds [30] is of utmost importance for basic research as well as for the pharmaceutical industry. Morphology as one phenotypic property was found as an indicator for the MΦ polarization state, where mouse and human MΦs induced similar morphological cell shape changes in response to intrinsic and extrinsic cues [30,31,32]. However, whether cell roundness is a universal and relevant phenotypic parameter to describe MΦ activation state remains elusive. Until now, only the extreme states of the MΦ polarization spectrum, pro- and anti-inflammatory, were characterized by defined morphological changes: M1(-like) are rather rounded, while M2(-like) are more elongated [30,31,32]. While these shape changes have been described for mouse (BMDMs) and human (MDMs) systems, a potential association of polarization state and cellular shape of IDMs had yet to be addressed. To assess cell shape changes by high-content imaging, we developed a cell roundness analysis tool (Z-score calculation) that reliably revealed polarization and reprogramming of IDMs treated with cpds and/or biological stimuli (Figure 2C,D, Figure 3, Figure 6A,C, and Figure S2B). In addition, we showed that primarily MDMs from several donors treated with biological or cpd stimuli have inter-donor variance in their heterogeneous cell shape response (Figure 3A,B). Moreover, cytotoxic cpd concentrations were reflected by a high Z-score, thereby erroneously pretending M1-like effects (Figure 2C). Therefore, in a potential phenotypic screening approach with cell roundness analysis as a read-out for MΦ-polarizing cpds, a parallel or sequential cytotoxicity assay would be warranted for the early exclusion of false-positive hits. Moreover, IDMs are suitable as a screening surrogate to MDMs with no heterogenous cpd-inducing cell shape response.

Consequently, we established an AI-fueled cell painting of human MDMs and IDMs to enable the early detection of cytotoxic effects in parallel with assessing the phenotypic polarization patterns of both MΦ systems. The principal component analyses of cell painting properties highlighted, for the first time, a feature-based comprehensive comparison of the phenotypic profiles of cpd- and biologically stimulated IDMs as well as the profiles of MDMs. Thus, feature-based profiling enables a description of different polarization fingerprints that potentially more realistically reflects the multidimensional spectrum of MΦ plasticity beyond the traditional dichotomous M1/M2 categorization (Figure S3A and Figure 4C). In this context, we established a ‘custom-made cell painting building block’ based on 1279 features that is already sufficient to describe the mentioned categorization (Figure 4C). Overall, the cell painting feature analysis is suitable for finding the inter-donor variance of MDMs and for dissecting the distinct MΦ polarization patterns when stimulated with known biological or chemical M1/M2 modulators (Figure S3A and Figure 4C). Thus, cell painting feature profiles broaden our existing framework about MΦ polarization, which is known to be induced by the modulation of several different pathways [30].

To identify and quantify MΦ (re-)polarization effects, we extended the feature-based cell painting analysis tool by training a ‘linear classifier’ that informs us about the polarization states across MΦ systems (IDMs and MDMs): M0, M1-like, and M2-like (Figure 4B). Importantly, the ‘linear classifier’ analysis enabled the identification of MΦ polarizing effects that were not found by the cell roundness analysis due to morphological heterogeneity (e.g., LPS or thiostrepton; Figure 5 and Figure 3A,B). Thus, cell types with no defined round or elongated cell shape can be studied based on their phenotypic profile by adapting our feature-based analysis tool. Moreover, by performing a potential high-throughput compound screen for MΦ-polarizing compounds, our analysis pipeline would inform us about a potential hit candidate, not only across different IDM lines but more importantly across the wide spectrum of donated MDMs. Additionally, a potential hit candidate can be characterized by comparing with fingerprints of cpds with known MoA, off-target activity, cytotoxicity, or bioactivity.

Using the trained ‘linear classifier’, we were able to address the relevance of standard morphology properties (area, roundness, width, length, and ratio of width to length) to indicate and discriminate different MΦ (re-)polarization states. In this context, we revealed that cell roundness is a relevant feature for the discrimination of M0 and M1(-like) states (Figure 7B). Surprisingly, neither cell roundness nor other standard morphological features are scored as relevant parameters to distinguish M1(-like) or M2(-like) states (Figure 7C). This is consistent for both MΦ systems and underlines the impact of morphological feature profiles to describe a cellular state.

To evaluate the established cell painting feature-based analysis tool and to test if the successful characterization of MΦ (re-)polarization would be restricted to this specific evaluation tool, we applied an orthogonal method by image-based DL. DL algorithms are powerful tools through their impact on drug discovery by accelerating the identification of effective drugs and their MoAs [87]. Therefore, we designed and developed together with ScreeningHub ValueData a DL-based cell painting analysis tool aligning in part with the training environment of the ‘linear classifier’: M0, M1-like, M2-like, and dead state. Identified by the ‘linear classifier’, the DL-based analysis tool confirmed the MΦ (re-)polarizing effects in MDMs and IDMs with high correlation scores (Figure 8B). In contrast to the cell roundness analysis, the DL-based analysis tool enables a quantification of the MΦ activation state effects and the identification of cytotoxicity-inducing cpd conditions. Thus, we designed and established two novel analysis tools, DL-fueled and feature-based, to generate an AI-fueled cell painting platform applicable for high-content and high-throughput screenings.

A future aim will be to implement our analysis tools as a novel and universal platform for the phenotypic screening of large cpd libraries to phenotypically characterize disease-relevant MΦ phenotypes (e.g., TAMs or profibrotic MΦs), study MΦ stimuli (e.g., profibrotic cocktails [88] or secreted cytokines), evaluate compound modulators (e.g., senescence or cell death inducers [89]), or compare cell painting signatures from MΦs of different origin (e.g., reprogrammed MΦs). In the long run, we aim to combine our analysis tools with multi-omics analyses [26] and functional read-outs like efferocytosis or phagocytosis capacity [19] to achieve the identification of an MΦ-polarizing compound in parallel with the transcriptional, efferocytotic, or proteomic changes that it induces. Moreover, IDMs are highly susceptible to genetic engineering [21], opening the potential of comparing chemical and genetic perturbations to support target deconvolution. Interestingly, a phenotypic signature, demonstrated by a predictive feature profile, can be a first novel indicator for the functional modulation of the cell system of choice. Therefore, combining high-content imaging with high-throughput screening opens a new avenue into unraveling complex biological processes by identifying potential modulators of the cellular state (e.g., shown for intestinal fibrosis [38]). Furthermore, we expect that our analysis tools find their utilization in early toxicology and safety assessment during hit finding and lead optimization. We speculate that the established cell painting analysis tools could accelerate hit triaging of selective MΦ-polarizing compounds, as demonstrated in other studies [43,90].

Overall, the increasing availability of image datasets from different cell systems (e.g., represented by a large publicly available reference dataset from the JUMP-CP (Joint Undertaking for Morphological Profiling-Cell Painting) consortium [91,92]) paired with information about chemical and genetical perturbation in a biological context will drive the development of more powerful DL models and feature extraction tools for drug discovery purposes.

## 4. Materials and Methods

### 4.1. Isolation of Human Monocytes from Leukapheresis Products and Differentiation Towards MΦs

Anonymized leukapheresis products from healthy human volunteers (donors) were obtained from Deutsches Rotes Kreuz (Ulm, Germany) in accordance with ethical standards and local regulations. Human peripheral blood mononuclear cells (PBMCs) were isolated using density gradient centrifugation using Ficoll-Paque Plus (GE Healthcare, Düsseldorf, Germany) and LeucosepTM (Greiner Bio-One, Frickenhausen, Germany). In this study, human monocytes were purified from frozen PBMC stocks using the EasySep^TM^ Human Monocyte Isolation Kit (19359RF, StemCell Technologies, Köln, Germany) according to the manufacturer’s RoboSep^TM^ protocol. In vitro differentiation of 1 × 10^6^ monocytes per well into human MΦs were cultured in 3 mL of complete DMEM GlutaMAX (31966-021, Gibco Thermo Fisher Scientific, Karlsruhe, Germany) supplemented with 10% heat-inactivated fetal calf serum (FCS; 10500064, Thermo Fisher Scientific, Karlsruhe, Germany; LOT10500-064) and 1% NEAA (11140050, Thermo Fisher Scientific, Karlsruhe, Germany) in the presence of 100 ng/mL recombinant human M-CSF (216-MC-500, R&D Systems, Wiesbaden, Germany) in Upcell 6-well plates (140685, Thermo Fisher Scientific, Karlsruhe, Germany) for 7 days. The quality control of RoboSep-isolated and purified monocytes and MΦs were performed by flow cytometry. The following donors were used in this study: CL119, CL090, CL091, CL4541, CL415, CL961, and CL414.

### 4.2. Differentiation of Human 201B7 iPSC Line CD14+- or CD34+-Derived Progenitors Towards MΦs

Cellartis human iPSC line 12 (ChiPSC12; cat. Y00285, Takara Bio Europe AB; Saint-Germain-en-Laye; France) and 201B7 [20] were cultured according to the manufacturer’s instructions in a feeder-free environment as a monolayer. The pluripotency of the 201B7 line was checked by flow cytometry. The maintenance and differentiation of the ChiPSC12 line towards MΦs is described in [19]. The ChiPSC12 line was used as a reference line to 201B7 and was therefore not primarily used in this study.

The differentiation protocol of human iPSCs from 201B7 into monocytes (CD14+-progenitors) towards MΦs was adapted from [18] and adjusted in the following steps of cytokine concentrations, progenitor expansion, medium usage, and cell density: Until the start of the mesodermal differentiation, the hiPSC (2500 cells/well/1.5 mL) were maintained in StemFit Medium Basic 03 (SFB-503, AmsBio, BH Alkmaar, Netherlands) supplemented with 100 ng/mL FGF (233-FB-025, R&D Systems, Wiesbaden, Germany) and 10 μM Y-27632 (10-2301, FOCUS Biomolecules, Plymouth Meeting, PA 19462, US) in a 6-well 1 h laminin iMatrix-511 (892012, Amsbio, BH Alkmaar, Netherlands) precoated plate (140675, Thermo Fisher Scientific, Karlsruhe, Germany) for 48 h. Every second day, the medium supplemented with FGF was refreshed for one week to reach 80% cell confluency. In total, 250 cells/well/1.5 mL were either seeded to start the mesodermal differentiation process or cryopreserved for long-term storage at −150 °C (50,000 cells/1vial/1 mL CryoStore (C2874, Sigma Aldrich, Schnelldorf, Germany)). To induce hematopoietic progenitors from day 7 in culture, the StemPro34^TM^ medium (10639011, Gibco Thermo Fisher Scientific, Karlsruhe, Germany) was used and supplemented with cytokines as described in [18]. Thereby, the concentration of M-CSF (rhM-CSF216-MC-500, R&D Systems, Wiesbaden, Germany) was increased to 100 ng/mL. In total, across three harvest rounds (one harvest round corresponds to one week), the monocytes were collected (harvested) through a 30 μM strainer (130-098-458, Miltenyi Biotec, Bergisch Gladbach, Germany) and differentiated into MΦs in complete DMEM GlutaMAX (31966-021, Gibco Thermo Fisher Scientific, Karlsruhe, Germany) supplemented with 10% heat-inactivated FCS (10500064, Gibco Thermo Fisher Scientific, Karlsruhe, Germany, LOT10500-064) and 1% NEAA (11140050, Gibco Thermo Fisher Scientific, Karlsruhe, Germany) in the presence of 100 ng/mL recombinant human M-CSF (216-MC-500, R&D Systems, Wiesbaden, Germany) in T75 flasks (5 × 10^6^ monocytes per T75 flask; 10537161, Falcon Corning, Kaiserslautern, Germany) for 7 days. The hematopoietic progenitors were kept to produce monocytes across the three harvest rounds being cultivated and expanded in StemPro34^TM^ medium (10639011, Gibco Thermo Fisher Scientific, Karlsruhe, Germany) supplemented with 1× GlutaMAX (A1286001, GibcoThermo Fisher Scientific, Karlsruhe, Germany), 100 ng/mL rh M-CSF (216-MC-500, R&D Systems, Wiesbaden, Germany), 50 ng/mL rh SCF (255-SC-050, R&D Systems, Wiesbaden, Germany), 10 ng/mL rh TPO (288-TPN-025, R&D Systems, Wiesbaden, Germany), 50 ng/mL rh IL3 (203-IL-050, R&D Systems, Wiesbaden, Germany), and 50 ng/mL rh FLT3L (308-FK-100, R&D Systems, Wiesbaden, Germany). The quality control of monocytes and MΦs were performed by flow cytometry analysis. Cellular imaging of the differentiation stages was conducted with an EVOS M5000 Imaging System (AMF5000, Thermo Fisher Scientific, Karlsruhe, Germany).

The differentiation protocol of human iPSCs from 201B7 into CD34+-progenitors was adapted from [19]. Details of the differentiation towards MΦs are described as follows: Across three harvest rounds, 10 × 10^6^ of CD34+-progenitors were frozen in 1 vial of 1 mL of CryoStore (C2874, Sigma Aldrich, Schnelldorf, Germany) for long-term storage at −150 °C. After thawing in a warm water bath, the CD34+-progenitors were washed once in complete DMEM GlutaMAX (31966-021, Gibco Thermo Fisher Scientific, Karlsruhe, Germany) supplemented with 10% heat-inactivated FCS (10500064, Gibco Thermo Fisher Scientific, Karlsruhe, Germany, LOT10500-064) and 1% NEAA (11140050, Gibco Thermo Fisher Scientific, Karlsruhe, Germany) in the presence of 100 ng/mL recombinant human M-CSF (216-MC-500, R&D), seeded at a density of 5 × 10^6^ per T75 flask (10537161, Falcon Corning, Kaiserslautern, Germany) in the respective medium and stored at 37 °C and 5% CO_2_. Every second day, fresh medium (5 mL per T75) and 100 ng/mL M-CSF (216-MC-500, R&D Systems, Wiesbaden, Germany) was added to the cells across two weeks. After one week in culture, the supernatant was collected and reseeded in 5 × 10^6^ of cells in a new T75 flask. The procedure of collecting and reseeding floating progenitors can be performed for two harvest rounds. The quality control of monocytes and MΦs was performed by flow cytometry. Cellular imaging of the differentiation stages was conducted using an EVOS M5000 Imaging System (AMF5000, Thermo Fisher Scientific, Karlsruhe, Germany).

### 4.3. Flow Cytometry

In total, 1 × 10^6^ cells/well were seeded in a 96-well cell repellent v bottom plate (651970, Greiner, Frickenhausen, Germany) in 200 μL of cold FACS blocking buffer (1× PBS,100100056, Gibco Thermo Fisher Scientific, Karlsruhe, Germany) supplemented with 2% FCS (10500064, Gibco Thermo Fisher Scientific, Karlsruhe, Germany) and 2 mM EDTA (UltraPure™ 0.5 M EDTA, pH 8.0; 15575020, Thermo Fisher Scientific, Karlsruhe, Germany) to determine a specific quality control panel of surface receptor markers using flow cytometry processing. After centrifugation for 5 min at 300× *g*, the cell pellet was resuspended in 50 μL of Fc blocking reagent (human, 130-059-901, Milentyi Biotech, Bergisch Gladbach, Germany) containing FACS blocking buffer (1:10 dilution) and incubated at 4 °C for 10 min in the dark. Based on the manufacturer’s instructions, 2.5 μL or 5 μL of the specific mouse anti-human antibody was prepared in a master mix reaction with its isotype as an additional control: CD14-Alexa488 (367129, Biolegend, Koblenz, Germany), CD206-APC (550889, BD, Heidelberg, Germany). CD34-PE (550761,BD, Heidelberg, Germany), CD163-BV786 (741003, BD, Heidelberg, Germany), CD80-BUV395 (565210, BD, Heidelberg, Germany), CD45-BV421 (563879, BD, Heidelberg, Germany), IgG1-Alexa488 (557782, Biolegend, Koblenz, Germany), IgG1-BV786 (563330, Biolegend, Koblenz, Germany), IgG1-BUV395 (563547, Biolegend, Koblenz, Germany), IgG1-BUV395 (563547, Biolegend, Koblenz, Germany), IgG1-BV421 (562438, Biolegend, Koblenz, Germany), IgG1-PE (555749, BD, Heidelberg, Germany), IgG1-APC (555751, BD, Heidelberg, Germany), TRA-1-81-PE (330708, Biolegend, Koblenz, Germany), SSEA-5-APC (355210, Biolegend, Koblenz, Germany), SSEA-1-BV789 (562277, BD, Heidelberg, Germany), SSEA-3-Alexa488 (560236, BD, Heidelberg, Germany), SSEA-4-BUV395 (563817, BD, Heidelberg, Germany), IgM k -PE (401611, Biolegend, Koblenz, Germany), IgG1 k -APC (400122, Biolegend, Koblenz, Germany), IgM k -BV786 (563837, Biolegend, Koblenz, Germany), IgG3k-BUV395 (563814, BD, Heidelberg, Germany), IgMk-Alexa488 (400811, Biolegend, Koblenz, Germany), and IgM k -BV421 (562704, BD, Heidelberg, Germany). An unstained control was prepared as well. Afterwards, 50 μL of antibody mix or FMO control was added to the 96-well plate, live–dead staining was enabled by the addition of 10 μL of FVS780/well (56388, BD, Heidelberg, Germany; 1:5000 dilution in FACS blocking buffer), and the plate was stored for 15 min at 4 °C. After washing 3 times with cold FACS blocking buffer, the samples were measured at the LSR Fortessa X-20 (BD, Heidelberg, Germany) and the expression levels were analyzed by FlowJo V10.5.3.

### 4.4. LDH Assay

The LDH assay was performed based on the manufacturer’s instructions of the LDH-Glo™ cytotoxicity assay (J2381, Promega, Walldorf, Germany) and adjusted in the following steps: The frozen supernatants from 0.2 × 10^6^ cells/well in a white opaque 96-well plate (353296, Falcon Corning, Kaiserslautern, Germany) were diluted 1:20 in LDH buffer (200 mM Tris-HCl (pH 7.3), 10% Glycerol, 1% BSA). No-cell, only-cell, and maximum LDH release (10% triton) conditions were used as internal controls and measured in triplicates.

### 4.5. Compound Treatment and Biological Stimulation

MDMs were harvested from 6-well Upcell plates (140685, Thermo Fisher Scientific, Karlsruhe, Germany) through the detachment of cells at room temperature for 1–2 h. In contrast, IDMs were harvested from the T75 flask (10537161, Falcon Corning, Kaiserslautern, Germany) by gentle scraping (Cell scraper, 83.3932, Sarsteadt, Nümbrecht, Germany) in 1× PBS (10010056, Gibco Thermo Fisher Scientific, Karlsruhe, Germany). MΦs were seeded in black 384-well Phenoplates (6057300302, Revvity, Hamburg, Germany) using a Multidrop^TM^ Combi Reagent Dispenser (5840300, Thermo Fisher Scientific, Karlsruhe, Germany) at a specific density (IDM: 9000 cells/well; MDM: 5000 cells/well) in 45 μL of complete DMEM GlutaMAX (31966-021, Gibco Thermo Fisher Scientific, Karlsruhe, Germany) supplemented with 10% FCS (10500064, Gibco Thermo Fisher Scientific, Karlsruhe, Germany, LOT10500-064) and 1% NEAA (11140050, Gibco Thermo Fisher Scientific, Karlsruhe, Germany) in the presence of 100 ng/mL recombinant human M-CSF (216-MC-500, R&D Systems, Wiesbaden, Germany). The seeded cells were stored overnight at 37 °C and 5% CO_2_. The cpd and biological stimuli (50 μL per condition/well) were prepared in complete DMEM in a stock concentration 10 times higher than the final one and transferred manually in at least quadruplicates into a Microplate deep well 384-storage plate (784201, Greiner, Frickenhausen, Germany). DMSO (final 0.1%; 12611S, CST, Frankfurt am Main, Germany) served as an internal negative control and was added to the biological stimuli as well if not otherwise indicated. The transfer of cpd or biological stimulation (5 μL per condition) from the Microplate deep well 384-storage plate (784201, Greiner, Frickenhausen, Germany) on the black 384-well Phenoplate (6057300302, Revvity, Hamburg, Germany) was performed semi-automatically with the CyBi-Well CyBio Automated Simultaneous Pipettor (69917-2, ARTISAN, Champaign, IL 61822, US). Lastly, the cell plate was stored for 24 h at 37 °C and 5% CO_2_. In case MΦ reprogramming was performed, the cells were stimulated with the biological stimuli during seeding, and after 24 h, the cpds were added for an additional 24 h. The following control biological stimuli were used alone or in combination in their indicated final concentration: 20 ng/mL rh IL4 (201-IL/CF 10 μg, R&D Systems, Wiesbaden, Germany), 20 ng/mL rh IL13 (213-IL/CF 25 μg, R&D Systems, Wiesbaden, Germany), 5 ng/mL LPS (L6143 Salmonella Sigma Aldrich, Schnelldorf, Germany), and 50 ng/mL TNFα (210-Ta-020/CF R&D Systems, Wiesbaden, Germany). The following cpds were used at different concentrations mentioned in the respective figure legend and serve as reference compounds [30]: thiostrepton (598226, Sigma Aldrich, Schnelldorf, Germany), bosutinib (PZ0192, Sigma Aldrich, Schnelldorf, Germany), fenbendazole (1269403, USP, Basel, Switzerland), alsterpaullone (A4847, Sigma Aldrich, Schnelldorf, Germany), etoposide (D1225, Selleckchem, Berlin, Germany), CA-074Me (205531, Millipore, Vienna, Austria), cytochalasin D (C8273, Sigma Aldrich, Schnelldorf, Germany), berberine chloride (PHR1502, Sigma Aldrich, Schnelldorf, Germany) and tetrandrine (S2403, Selleckchem, Berlin, Germany).

### 4.6. Cell Painting and High-Content Imaging

The cell painting assay was adapted by the manufacturer’s instructions from the PhenoVue Cell Painting Kit containing 6 multiplexing cellular dyes (PING12, Revvity, Hamburg, Germany) and adjusted in the workflow in the final concentration of the staining reagents and the image acquisition parameters in the confocal Opera Phenix^TM^ high-content screening device (HH14000000, Revvity, Hamburg, Germany). The dyes were diluted in 2× PhenoVue Dye Dilutent (0.22 μM filtered) as follows: PhenoVue 641 Mitochondrial stain 1mM (1:16,000), PhenoVue Hoechst 33342 nuclear stain 1 mg/mL (1:1600), PhenoVue Fluor 488-concanavalin A 2 mg/mL (1:800), PhenoVue 512 nucleic acid stain 5 mM (1:1500), PhenoVue Fluor 555-WGA 0.15 mg/mL (1:200), and PhenoVue Fluor 568-Phalloidin 6.6 μM (1:400). Specifically, the PhenoVue Fluor 488-concanavalin A dye was titrated to reduce the spectral overlap with the PhenoVue 512 nucleic acid stain that was acquired in one channel due to no separate emission filter (used emission filters: 435–480 nm; 435–550 nm; 500–550 nm; 570–630 nm; 650–760 nm) capacity. The procedure of fixing, permeabilizing, staining, and washing the MΦs was performed semi-automatically using the Multidrop^TM^ Combi Reagent Dispenser (5840300, Thermo Fisher Scientific, Karlsruhe, Germany) and BioTek^®^ 50^TM^ TS Microplate Washer (Millipore, Vienna, Austria): First, the medium from pretreated MΦs in the 384-black Phenoplates was removed up to 20 μL/well and 50 μL/well of PhenoVue 641 mitochondrial stain solution was added. After 30 min of incubation at 37 °C and 5% CO_2_, the cells were fixed with 2.0% PFA (16% stock; 20 μL/well) and stored in the dark at room temperature for 30 min. After removing PFA by washing three times with 1× PBS, the cells were permeabilized with 0.1% tritonX-100 (20 μL/well) and incubated in the dark at room temperature for an additional 20 min followed by a three times 1× PBS wash. Then, the cells were stained with the remaining 5 dyes (20 μL/well) and incubated in the dark at room temperature for 30 min. Finally, the cells were washed again three times with 1× PBS, and 20 μL/well of 1× PBS was added to the cells, followed by sealing the plate. Images were acquired at binning mode 2 at 40× (minimum of 10 fields per imaged well) and 20× magnification (minimum of 5 fields per imaged well) in water immersion in the confocal Opera Phenix^TM^ high-content screening device using Harmony software5.2 (Revvity, Hamburg, Germany). Further imaging parameters were used: Autofocus: two peak defaults; objective: 20× water; NA 1.0 or 40× water NA 1.1; confocal optimal mode and camera ROI: 2160 × 2160 px. To increase the fluorescence intensity and preferably decrease the spectral crosstalk, the emission and excitation parameters were set as follows based on the used magnification for IDMs and MDMs systems: 1. For 20× magnification: Hoechst 33342 (excitation: 405 nm; emission: 435–480 nm; time: 700 ms, power: 100%, height: −5.0 μM), Alexa 488 (excitation: 488 nm; emission: 500–550 nm; time: 40 ms, power: 20%, height: −6.0 μM), Alexa 647 (excitation: 640 nm; emission: 650–760 nm; time: 40 ms, power: 50%, height: −5.0 μM), Alexa 568 (excitation: 561 nm; emission: 570–630 nm ; time: 60 ms, power: 100%, height: −6.0 μM); 2. For 40× magnification: Hoechst 33342 (time: 700 ms, power: 100%, height: 1.0 μM), Alexa 488 (time: 40 ms, power: 20%, height: 0 μM), Alexa 647 (40 ms, power: 50%, height: 0 μM), Alexa 568 (time: 60 ms, power: 100%, height: 0 μM). These described settings were tested in single and multiplexed dye assays across days because 384-well plates were imaged immediately or after 3 days of being stored at 4 °C. The images acquired in Harmony software of Opera Phenix^TM^ were processed for data analysis in Signals Image Artist (SImA1.3) software from Revvity (Hamburg, Germany).

### 4.7. Cell Roundness Analysis

The image acquisition parameters are indicated in 5.6. For image data analysis, we used Signals Image Artist software (SImA1.3) purchased from Revvity (Hamburg, Germany). Each ’Input Image’ (Channel group:1; Sequences: ALL) was flatfield-corrected (advanced). Each population was predefined by ‘Find Nuclei’ (Channel: Hoechst33352; Method: B; ROI: none) and ‘Find Cytoplasm’ (Channel: Alexa488; Method: B). Border objects were not considered. To determine the cell roundness, the ‘Calculate morphology properties’ building block was selected (Population: Nuclei; Method: standard; Region: Cell). The processed cell roundness values were normalized to the vehicle control DMSO by using the following Z-score equation: Z-score = cell roundness (condition)- mean cell roundness (DMSO)/standard deviation cell roundness (DMSO). A value of >0 was used as an indicator for a more rounded cell (M1(-like)), whereby a value of <0 was an indicator of an elongated morphology (M2(-like)). To exclude compound-induced cell cytotoxicity, the cell number was determined as ‘Nuclei-Number of Objects’, informing us about the DAPI-stained nuclei.

### 4.8. Feature-Based Cell Painting Analysis

For image data analysis, we used Signals Image Artist software (SImA1.3) purchased from Revvity (Hamburg, Germany). The following channels were used in the feature-based analysis: Alexa488, Alexa568, Alexa647, and Hoechst33342. The image acquisition parameters are indicated in 5.6. Each ’Input Image’ (Channel group:1; Sequences: ALL) was flatfield-corrected (advanced). Each population was predefined by ‘Find Nuclei’ (Channel: Hoechst33352; Method: B; ROI: none) and ‘Find Cytoplasm’ (Channel: Alexa488; Method: B). Border objects were not considered. For using the ‘preset cell painting building block’ (4710 features), we followed the instructions (Harmony Software Manual—Opera Phenix) from the Revvity (Hamburg, Germany): Method: default regions; channels: Alexa488, Alexa568, Alexa647, Hoechst33342; SER scale: 2; property set: extensive; regions: nucleus (inner border: 100%; outer border: 50%), membrane (inner border: 10%; outer border: 0%), cytoplasm (inner border: 50%; outer boarder: 0%), ring (inner border: 50%; outer border: 30%), cell (inner border: 100%; outer border: 0%). To phenocopy the ‘preset cell painting building block’, we calculated the texture, intensity, and morphology properties in all four channels (Alexa488, Alexa568, Alexa647, and Hoechst33342) over three regions (cytoplasm, nucleus, and full cell), reaching 1279 features in total (‘custom-made cell painting building block’). All three properties were defined by the following feature selections: texture properties (SER and Gabor features normalized by kernel), intensity properties (standard features), and morphology properties (standard features and STAR features with sliding parabola combined with SER features normalized by kernel). We extracted the properties per single object and used the mean of each property per well for further processing. We combined the ‘custom-made cell painting building block’ with the method for object classification using the PhenoLOGIC™ linear classifier algorithm ([75], named in the study as ‘linear classifier’) from Revvity (Hamburg, Germany). In detail, the method ’linear classifier’ was used for ‘Select Population’ and trained on 100 cells per output population (M0, M1-like, and M2-like) from randomly selected 20× magnified fields of wells from images of 384-well plates based on the instructions in ‘Harmony Software Manual—Opera Phenix’ and ‘Image Analysis Guide’ from Revvity (Hamburg, Germany). The definition of the output population was dependent on the generated model as indicated by Figure 5A. The information about cytotoxic effects was incorporated by the cell numbers. To address the impact of morphology properties in the trained model 2 ‘linear classifier’ (Figure 7A), we removed in the ‘Select Population’ building block all ‘standard morphology properties’ (area, cell roundness, width, length, and ratio of width to length) or only the ‘Cell Roundness’ feature. Mean per well %M2(-like) values and %M1(-like) values were normalized by subtraction of the average mean per well %M2(-like) or %M1(-like) values of the respective DMSO control on the same plate and then the normalized mean %M2(-like)—%M1(-like) values calculated for each treatment. For the PCAs, the ‘preset cell painting building block’ features and the ‘custom-made cell painting building block’ features were centered by the subtraction of the mean and scaled by dividing by each features’ standard deviation before calculating the first two principal components accounting for the highest amount of variance in the dataset. Figures were generated using R-Project4.2.2 and RStudio 2022.12.05+353 ‘Elsebeth Geranium’ using the ggplot2 and ggrepel packages and edited using Adobe Illustrator2022 version 26.4.

### 4.9. Deep Learning-Based Cell Painting Analysis

ScreeningHub provided by ValueData GmbH is an AI-based screening platform using deep learning for cell-based phenotypic drug discovery to, inter alia, automate the detection and classification of MΦs into four distinct categories: M0, M1-like, M2-like, and dead cells. The 40× cell painting images of 201B7 IDMs from several 384-well plates treated with the same treatment conditions as ‘linear classifier model 2′ were processed through the model, which utilized convolutional neural networks (CNNs) for object detection. In contrast to the feature-based analysis, a dead category was trained using images treated with staurosporine 1 μM (Sigma Aldrich, Schnelldorf, Germany). The CNN-based model was designed to accurately locate individual cells and classify them according to their specific phenotype. The input images underwent a series of preprocessing steps to enhance the quality and consistency of the data before being analyzed by the AI model. These steps were designed to optimize the images for accurate detection and classification, ensuring that the relevant features of each cell were emphasized for the DL algorithm. For each detected cell, the model assigned a label corresponding to one of the four cell types: M0, M1-like, M2-like, or dead. Additionally, the model generated a confidence score for each classification, which indicated the probability associated with the predicted cell type. Cells with a confidence score below a threshold of 40% were excluded from further analysis. The classified cells for each experimental condition were aggregated to analyze the distribution of cell types, providing a detailed examination of how various treatments affected macrophage polarization. For every well, the total number of cells detected, the ratio of M0, M1-like, M2-like, and dead cells, and the average confidence score for each class were calculated. Results across replicate wells for the same condition were averaged to ensure consistency and to minimize the impact of potential experimental variability. The analysis results (Figure 8 and Figure S5A) were automatically plotted using ScreeningHub and edited using AdobeIllustrator2022 version 26.4.

### 4.10. Statistical Analysis

Statistics were performed with GraphPad Prismv10.1.2 (GraphPad Software, San Diego, CA, USA). The data are reported as the means of at least three independent experiments (specific number of replicates mentioned in the figure legends). Statistically significant differences were calculated using a two-way ANOVA with Tukey’s multiple comparisons test or Student’s *t*-test (* *p* < 0.05, ** *p* < 0.01, *** *p* < 0.001, and **** *p* < 0.0001). The data from flow cytometry are depicted as percentages. Biological replicates (individual experiments or MΦ donors) were measured independently, and technical replicates were generated out of biological ones. Correlation coefficients were defined by a Pearson correlation.

## 5. Conclusions

In this study, we established the AI-fueled cell painting of human IDMs and MDMs and demonstrated that the magnitude of cpd-induced MΦ (re-)polarization can be identified and quantified by feature- or DL-based analyses with the parallel detection of toxic effects with a high correlation score. The PCA of the respective cell painting fingerprints informed us about subtle phenotypes of IDMs and MDMs induced by different biological or chemical stimuli and impressively reflected the compelling spectrum of MΦ plasticity compared with the traditional and rather simplistic M1/M2 dichotomy. Surprisingly, using feature-based cell painting analysis, we found that the cell roundness feature or standard morphology properties (area, roundness, width, length, and ratio of width to length) were not scored as relevant ones to discriminate between M1(-like) and M2(-like) polarization states. However, cell roundness as a morphology feature is relevant for distinguishing between unpolarized and pro-inflammatory MΦs. In addition, we highlighted the potential of IDMs as a versatile screening surrogate for the phenotypically more heterogeneous MDMs. We expect that our novel deep learning and feature extraction analysis tools to generate and analyze cell painting imaging data will enhance hit identification and triaging in high-content phenotypic screens to identify modulators of disease-relevant phenotypes in MΦ biology and will open new avenues for a better understanding of MΦ (re-)polarization.

## Data Availability

The imaging data presented in this study are available in SImA. Access to the data can be obtained by email to the corresponding author.

## Supplementary Materials

The following supporting information can be downloaded at: https://www.mdpi.com/article/10.3390/ijms252212330/s1.
