# Supplementary material for: Insights into the Identification of iPSC- and Monocyte-Derived Macrophage-Polarizing Compounds by AI-Fueled Cell Painting Analysis Tools
Source: Int J Mol Sci. 2024 Nov 17;25(22):12330. doi: 10.3390/ijms252212330 (PMC11595184; doi:10.3390/ijms252212330)
Supplement: Supplementary file 1 [file ijms-25-12330-s001.zip › SUPPLEMENTARY INFORMATION_proofreading_completed.pdf]

Insights into the identification of iPSC- and monocyte-derived macrophage-polarizing compounds by AI-fueled cell painting analysis tools

SUPPLEMENTARY INFORMATION

Supplementary Figure

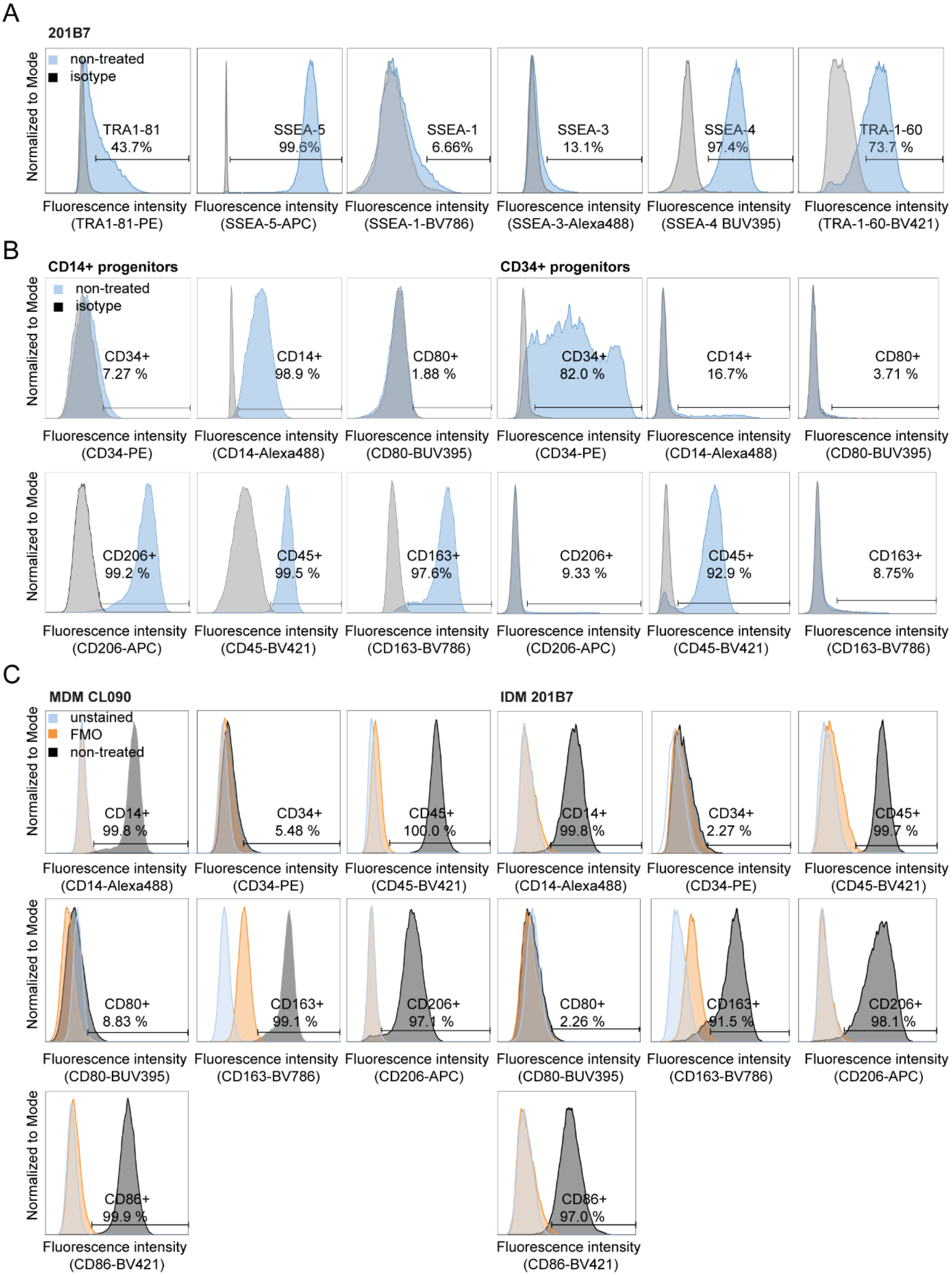

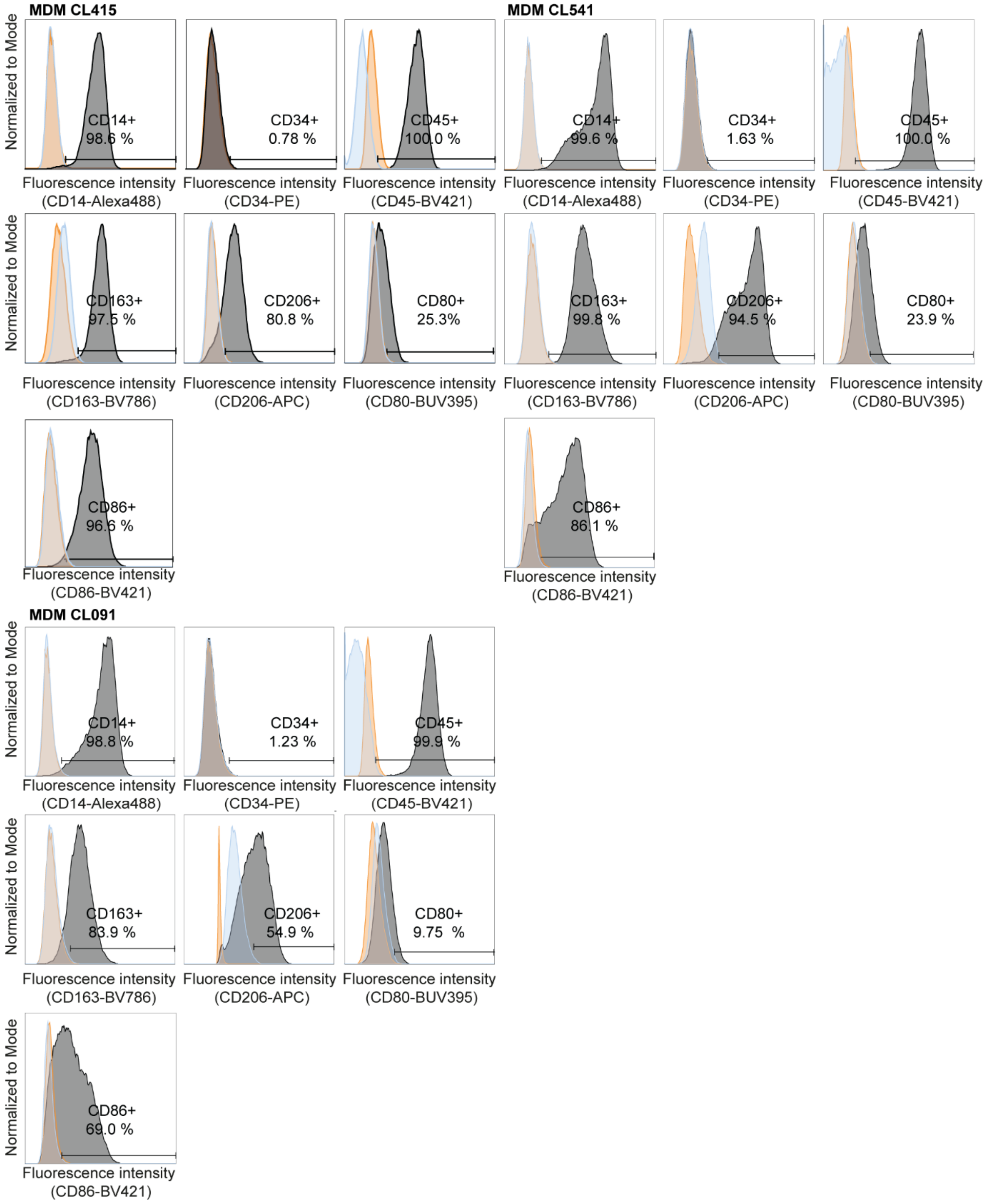

Figure S1. Surface marker expression for quality control of differentiation stages of 201B7, CD14+ or CD34+ progenitors and MΦs. **(A)** Flow cytometry analysis of pluripotency (TRA1-81, SSEA-5, SSEA-1, SSEA-3, SSEA-4 and TRA-1-60 expression) of hiPSC 201B7 line (in %; n=3; gating is based on 1% isotype control). **(B)** Flow cytometry analysis of specific surface marker expression (lymphocyte marker CD45, monocytic lineage marker CD14, hematopoietic stem cell marker CD34, macrophage markers CD206, CD163 and CD80) of CD14+- or CD34+- progenitors (in %; n=3; gating is based on 1% isotype control).

(C) Flow cytometry analysis of specific surface marker expression (lymphocyte marker CD45, monocytic lineage marker CD14, hematopoietic stem cell marker CD34, macrophage markers CD206, CD163, CD86 and CD80) of MΦs (IDMs and MDM donors CL090, CL541, CL415, CL091; in %; n=3; gating is based on 1% isotype control).

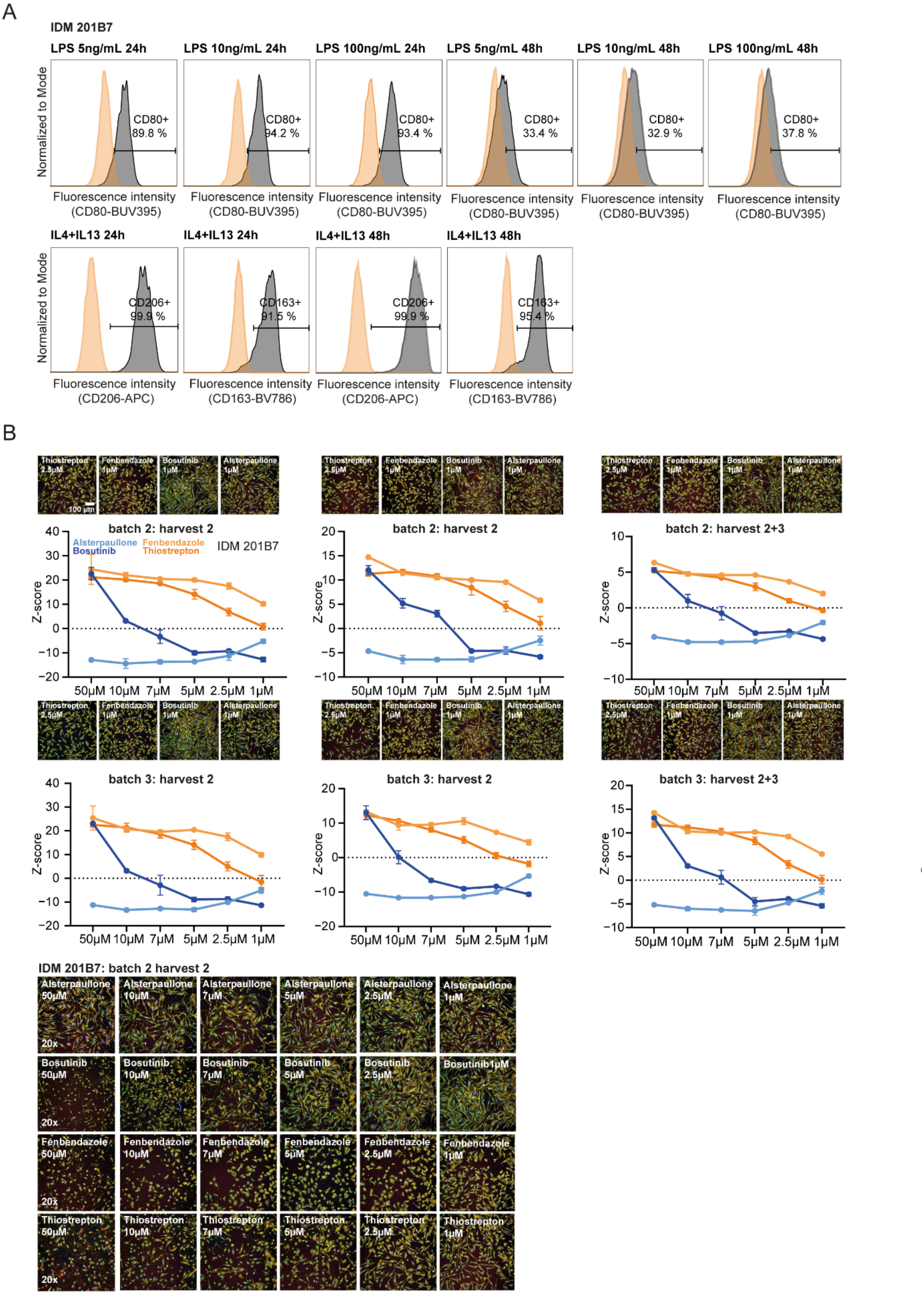

Figure S2. Time- and dose-dependent analysis of compound treated and biological stimulated IDMs. **(A)** Flow cytometry analysis of specific surface MΦ marker expression (macrophage markers CD206,

CD163 and CD80) of LPS or IL4+IL13 treated IDMs at 24h or 48h (in %; n=3; gating is based on 1% isotype control). **(B)** Dose-response analysis of Z-score calculated cell roundness analysis. Data are depicted as mean±SD. Representative high-content Opera Phenix™ confocal images of cell painted (PhenoVue Kit: PhenoVue 641 Mitochondrial stain, PhenoVue Hoechst 33342 Nuclear stain, PhenoVue Fluor 488-Concanavalin A, PhenoVue 512 Nucleic Acid stain, PhenoVue Fluor 555-WGA and PhenoVue Fluor 568-Phalloidin) IDMs treated with illustrated conditions for 24h. Each condition (4 wells; 5 fields per well) was imaged in one 384-well plate (20× magnification). IDMs were generated from different frozen CD34+-progenitor stocks (harvest) across different progenitor production rounds (batch). Pooled batch 2 and 3 is indicated as batch 2+3.

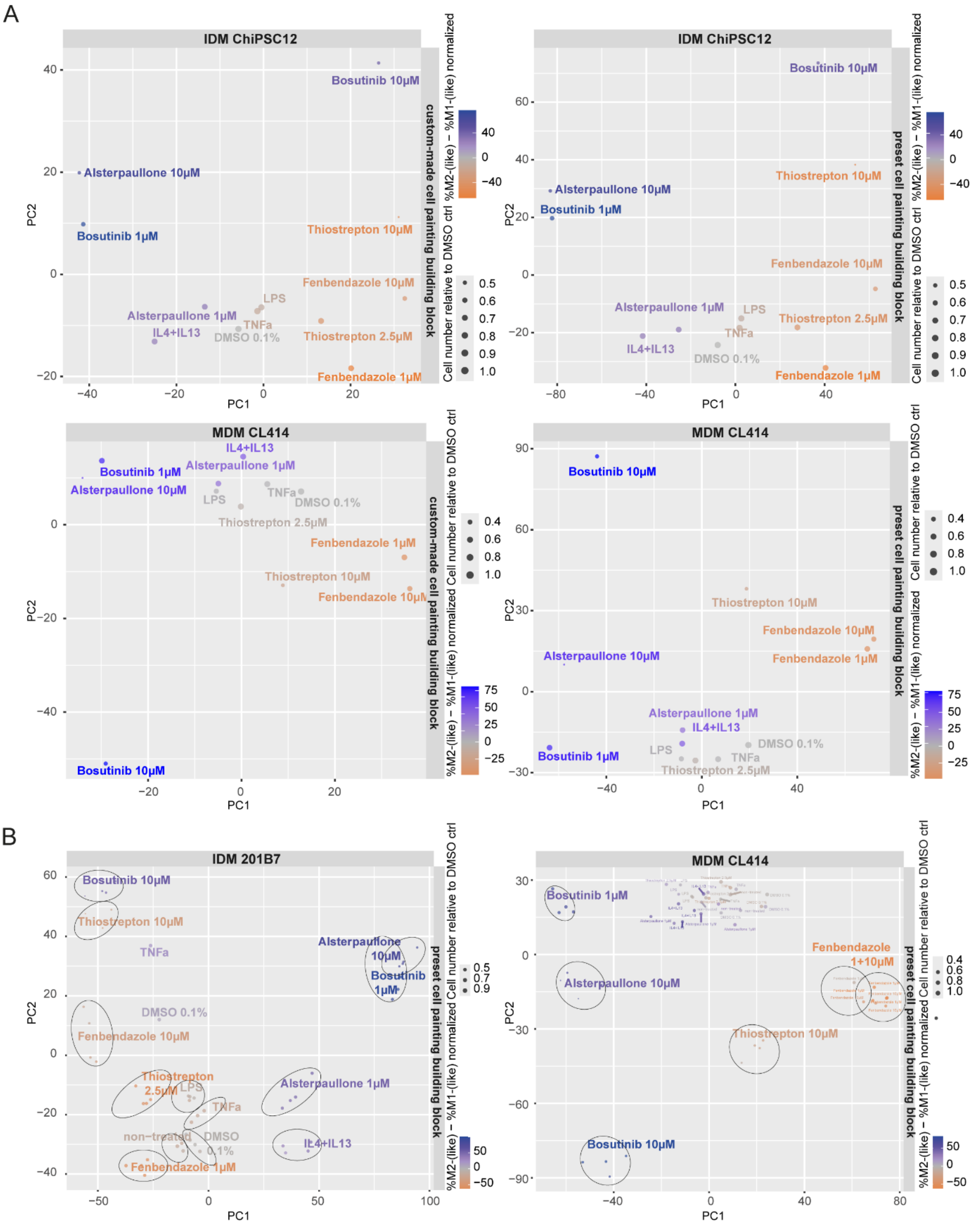

Figure S3. Cell painting feature profiles of compound and biological stimulated MΦs. **(A)** Principal component analysis of the 'Preset cell painting building block' and the 'custom-made cell painting building block' features indicating cpd and biological stimulated ChiPSC12 IDMs and MDM donor CL414 for 24h. Each datapoint indicates one well of total 4 replicate wells (5 fields per well) per condition. 'Preset cell painting building block' based on 4710 and 'custom-made cell painting building

block' based on 1279 features. **(B)** Principal component analysis of 'preset cell painting feature building block' features indicated cpd and biological stimulated 201B7 IDMs and MDM CL961 for 24h. Each datapoint indicates the individual well (5 fields per well; 4 wells) per condition. 'Preset cell painting building block' based on 4710 features.

Insights into the identification of iPSC- and monocyte-derived macrophage-polarizing compounds by AI-fueled cell painting analysis tools

A

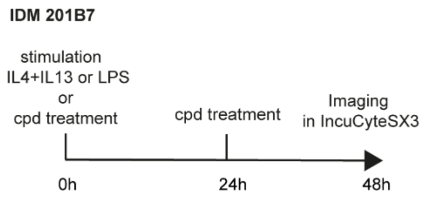

DMSO 0.1%_IDM_B3_3.mp4

non-treated_IDM_B2_3.mp4

LPS 5ngmL_IDM_F3_3.mp4

Staurosporine 1µM_IDM_B12_3.mp4

Bosutinib 1µM_IDM_B5_3.mp4

Thiostrepton 2.5µM_IDM_B4_3.mp4

LPS+Bosutinib_IDM_F11_5.mp4

IL4+IL13 20ngmL_IDM_F2_3.mp4

IL4+IL13+Thiostrepton_IDM_F5_3.mp4

B

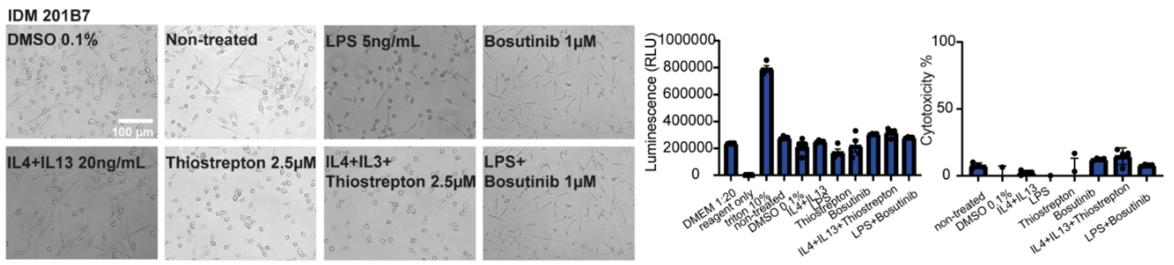

C

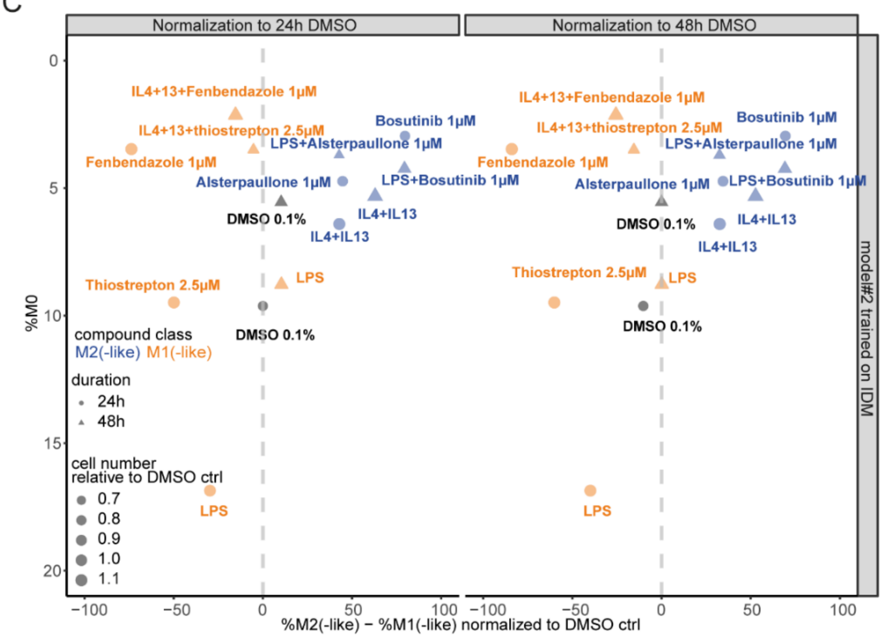

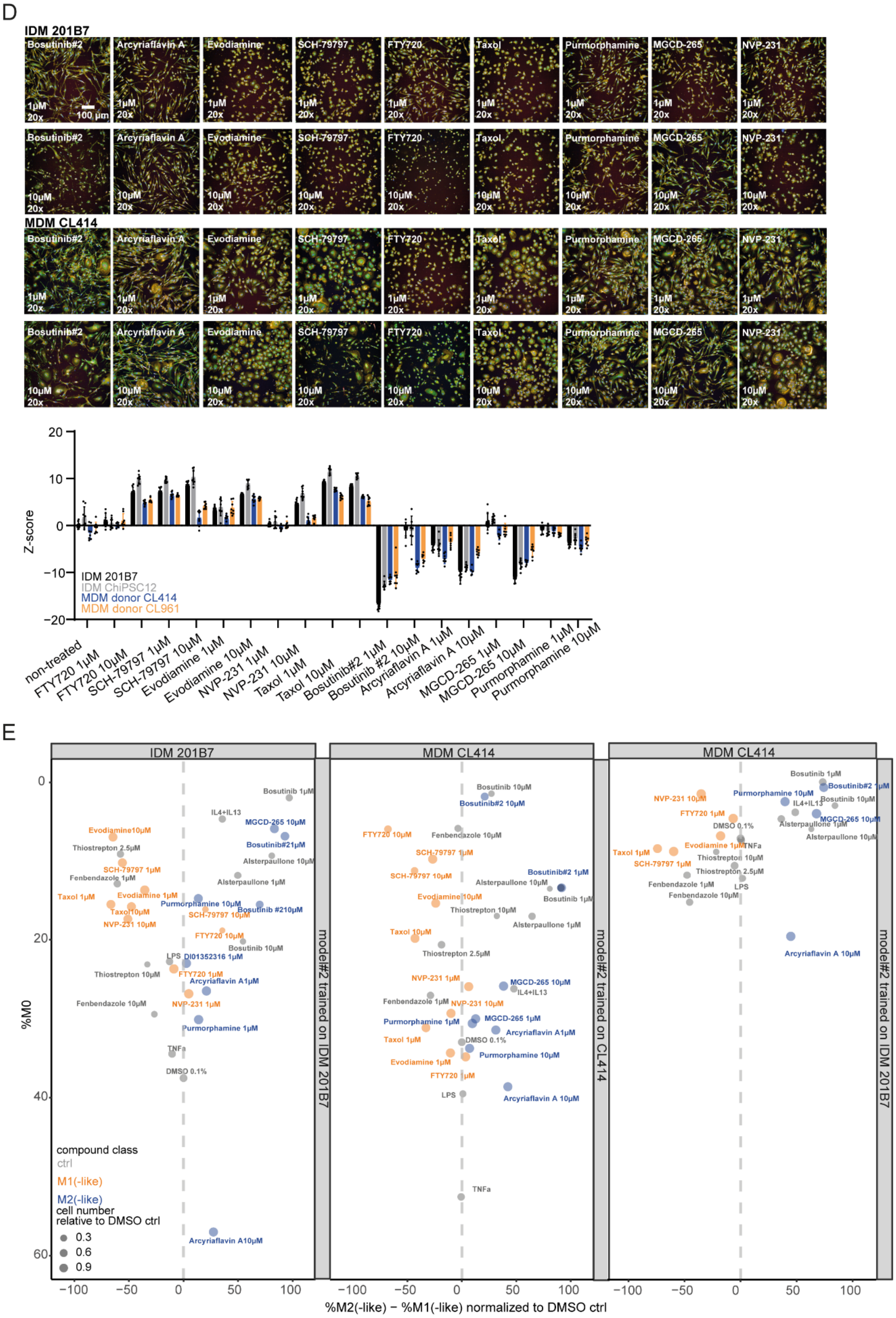
D
IDM 201B7
MDM CL414
Bosutinib#2
Arcyriaflavin A
Evodiamine
SCH-79797
FTY720
Taxol
Purmorphamine
MGCD-265
NVP-231
100 µm
Z-score
IDM 201B7
IDM ChiPSC12
MDM donor CL414
MDM donor CL961
non-treated
FTY720 1µM
FTY720 10µM
SCH-79797 1µM
SCH-79797 10µM
Evodiamine 1µM
Evodiamine 10µM
NVP-231 1µM
NVP-231 10µM
Taxol 1µM
Taxol 10µM
Bosutinib#2 1µM
Bosutinib #2 10µM
Arcyriaflavin A 1µM
Arcyriaflavin A 10µM
MGCD-265 1µM
MGCD-265 10µM
Purmorphamine 1µM
Purmorphamine 10µM
E
IDM 201B7
MDM CL414
MDM CL414
model#2 trained on IDM 201B7
model#2 trained on CL414
model#2 trained on IDM 201B7
%M0
%M2(-like) − %M1(-like) normalized to DMSO ctrl
compound class
ctrl
M1(-like)
M2(-like)
cell number relative to DMSO ctrl
0.3
0.6
0.9

F

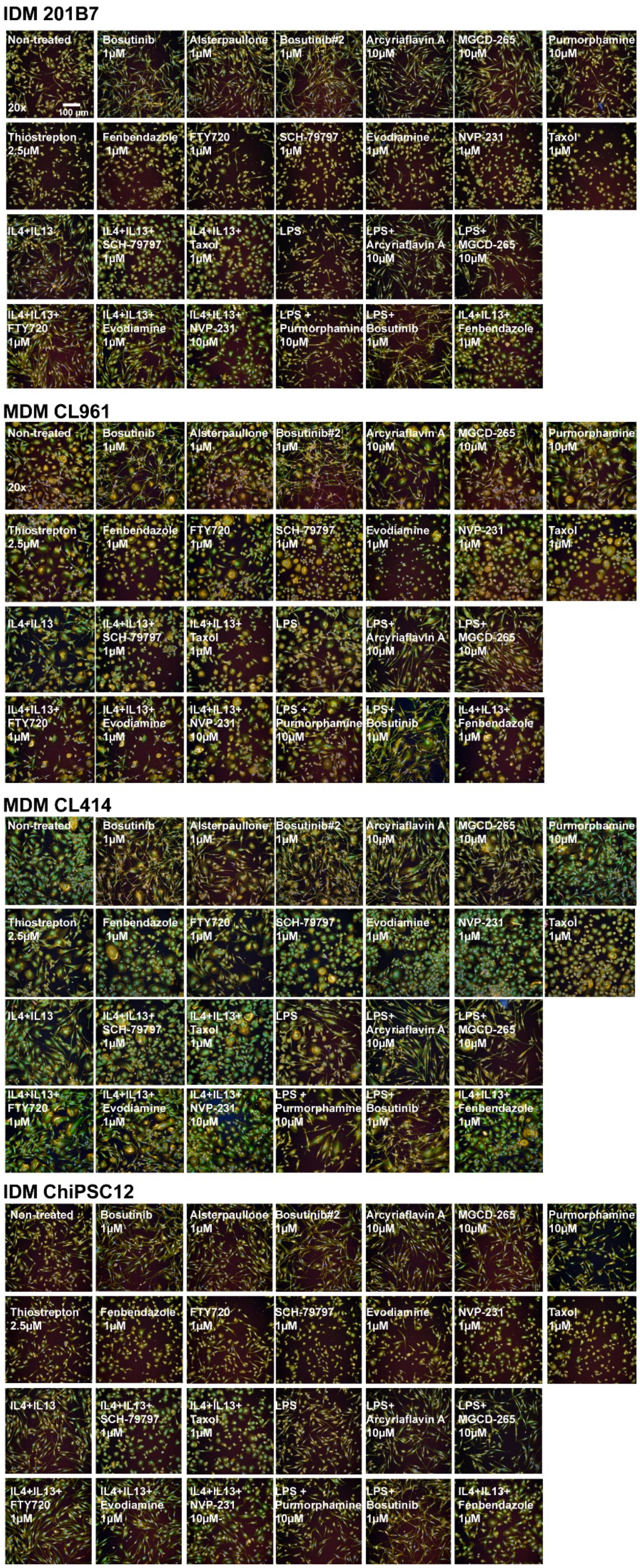
IDM 201B7
Non-treated
20x
100 µm
Bosutinib 1µM
Alsterpaullone 1µM
Bosutinib#2 1µM
Arcyriaflavin A 10µM
MGCD-265 10µM
Purmorphamine 10µM
Thiostrepton 2.5µM
Fenbendazole 1µM
FTY720 1µM
SCH-79797 1µM
Evodiamine 1µM
NVP-231 1µM
Taxol 1µM
IL4+IL13
IL4+IL13+ SCH-79797 1µM
IL4+IL13+ Taxol 1µM
LPS
LPS+ Arcyriaflavin A 10µM
LPS+ MGCD-265 10µM
IL4+IL13+ FTY720 1µM
IL4+IL13+ Evodiamine 1µM
IL4+IL13+ NVP-231 10µM
LPS + Purmorphamine 10µM
LPS+ Bosutinib 1µM
IL4+IL13+ Fenbendazole 1µM
MDM CL961
MDM CL414
IDM ChiPSC12

Figure S4. Reprogramming of compound and biological stimulated MΦs. **(A)** Time-course analysis of indicated M1- and M2-like cpds single or combined with biological stimuli treated 201B7 IDMs in the IncuCyte SX3 (0h to 48h; imaging every h; 10x magnification). Schematic and representative videos of one field of the whole well per indicated condition. Staurosporin 1μM treatment served as cytotoxic control. **(B)** Representative brightfield images at 20× magnification of 201B7 IDMs treated with indicated compounds and/or biological stimulation. LDH assay results of the treated conditions: data are depicted as mean±SD from four replicates per condition. **(C)** SImA linear classifier quantification of IDMs trained on IDM model 2 from Figure 5A. DMSO 0.1%-normalized values correspond to a mean of 4 wells imaged at 20× magnification (5 fields per well). DMSO-normalized %M2(-like) - %M1(-like) values are listed in Table S3. Treatment conditions used from Figure 6A. Normalization was performed to either 24h DMSO or 48h DMSO treatment. **(D)** Representative high-content Opera Phenix™ confocal images of cell painted (PhenoVue Kit: PhenoVue 641 Mitochondrial stain, PhenoVue Hoechst 33342 Nuclear stain, PhenoVue Fluor 488-Concanavalin A, PhenoVue 512 Nucleic Acid stain, PhenoVue Fluor 555-WGA and PhenoVue Fluor 568-Phalloidin) IDMs (ChiPSC12 and 201B7) and two MDM donors (CL961 and CL414) with indicated M1- and M2-like cpds single-treated or combined with biological stimuli (4 wells per condition; 5 fields per well; 20× magnification) for 24h. Z-score calculation for cell roundness analysis: data are depicted as mean±SD from 20× magnification (from two independent 384-well plates). **(E)** SImA linear classifier quantification of IDMs and MDMs (donor CL414) trained on IDM model 2 from Figure 5A and MDMs trained on their own. DMSO 0.1%-normalized values correspond to a mean of 4 wells imaged at 20× magnification (5 fields per well). DMSO-normalized %M2(-like) - %M1(-like) values are listed in Table S4 and S5. Treatment conditions are indicated. **(F)** Representative high-content Opera Phenix™ confocal images of cell painted (PhenoVue Kit: PhenoVue 641 Mitochondrial stain, PhenoVue Hoechst 33342 Nuclear stain, PhenoVue Fluor 488-Concanavalin A, PhenoVue 512 Nucleic Acid stain, PhenoVue Fluor 555-WGA and PhenoVue Fluor 568-Phalloidin) IDMs (ChiPSC12 and 201B7) and two MDM donors (CL961 and CL414) with indicated M1- and M2-like compounds single-treated or combined with biological stimuli (4 wells per condition; 5 fields per well; 20× magnification) for 24h. Corresponding Z-score calculation is indicated in Figure 6C.

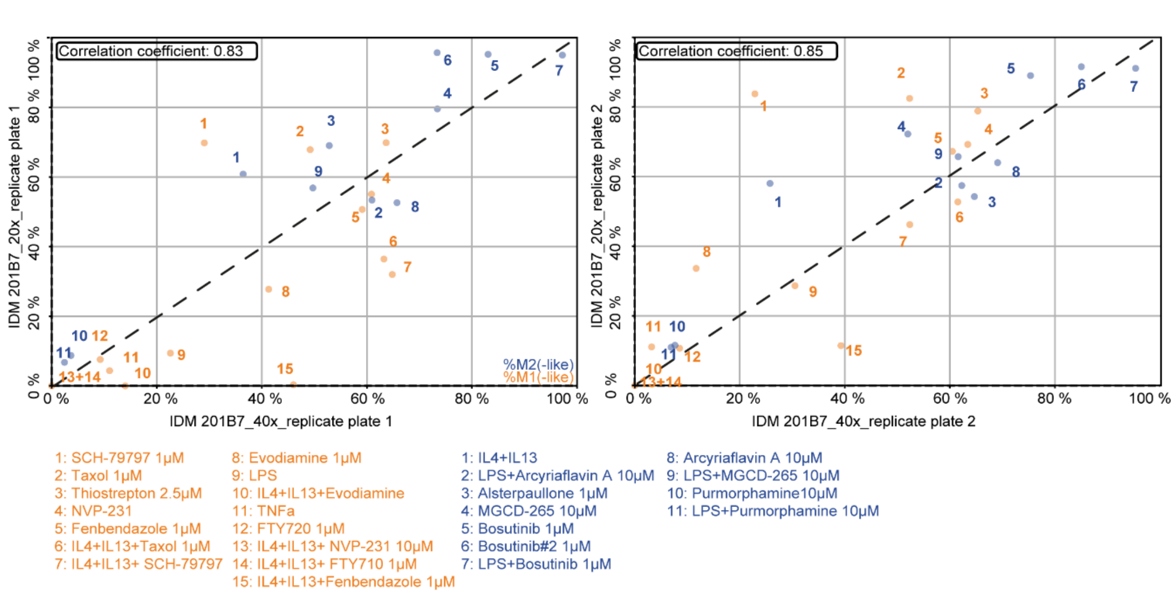

Figure S5. Deep learning-based determination of MΦ (re)-polarization state. **(A)** Scatter plot of cell ratios (% M1(-like) and M2(-like) proportion) of the DL-based analysis of 24h cpd and biological stimulated 201B7 IDMs from two replicate 384-well plates trained on IDM DL-model 2 (40× magnification). DMSO 0.1%-normalized values correspond to a mean of 4 wells imaged at 40×magnification (10 fields per well) or 20× magnification (5 fields per image). Correlation coefficient is calculated by Pearson correlation.

Supplementary Tables

The supplementary tables (Table S1-S7) can be downloaded at:

Supplementary Videos

The supplementary videos belonging to FigureS4 can be downloaded at:
